# Toronto’s Supervised Consumption Sites and Local Crime

**DOI:** 10.1001/jamanetworkopen.2025.45352

**Published:** 2025-11-25

**Authors:** Dimitra Panagiotoglou, Jihoon Lim, Geoffrey Ingram, Mariam El Sheikh, Imen Farhat, Xander Bjornsson, Maximilian Schaefer

**Affiliations:** 1 Department of Epidemiology, Biostatistics and Occupational Health, McGill University, Montreal, Québec, Canada

## Abstract

**Importance:**

Beginning in August 2017, 9 overdose prevention sites and supervised consumption sites (OPS/SCS) began operating in Toronto, Canada. Following years of community pushback stating that these sites increased local crime and disorder, they were closed in March 2025.

**Objective:**

To examine the association between OPS/SCS and local crime and disorder.

**Design, Setting, and Participants:**

This ecological cohort study used Toronto Police Service data to compare crime incidence before and after OPS/SCS implementation using an interrupted time series study design. Analysis was restricted to crimes that occurred within city boundaries between January 1, 2014, and June 30, 2025.

**Main Outcomes and Measures:**

The study used monthly event counts of all assaults, auto thefts, break and enters, robberies, thefts over $5000, bicycle thefts, and thefts from motor vehicles as the 7 outcomes. Incidence within 400 m of the geolocation of each OPS/SCS before and after implementation were compared and results were pooled for population-level estimates.

**Results:**

Within 400 m (approximately a quarter mile), OPS/SCS implementation was associated with increases in break and enters (49.88%; 95% CI, 27.03% to 76.84%), and to a lesser extent, thefts from motor vehicles (20.03%; 95% CI, −0.63% to 44.99%). However, monthly trends for break and enters (−1.19%; 95% CI, −1.71% to −0.68%), robberies (−1.32%; 95% CI, −1.93% to −0.70%), thefts over $5000 (−1.48%; 95% CI, −2.45% to −0.50%), bicycle thefts (−1.82%; 95% CI, −2.93% to −0.68%), and thefts from motor vehicles (−1.30%; 95% CI, −2.18% to −0.42%) declined. Site-specific results revealed some OPS/SCS were associated with increases in crime while most were not.

**Conclusions and Relevance:**

This ecological cohort study found that the association between Toronto’s OPS/SCS and crime was generally neutral to beneficial. OPS/SCS were associated with increases in break and enters, and to a lesser extent thefts from motor vehicles, immediately postimplementation. Incidents of crimes declined with time.

## Key Points

**Question:**

What was the association between overdose prevention sites and supervised consumption sites (OPS/SCS) and local crime in Toronto?

**Findings:**

This ecological cohort study found OPS/SCS were associated with immediate increases in break and enters, but trends in outcomes declined per month, while trends in assaults, robberies, thefts over $5000, bicycle thefts, and thefts from motor vehicles also decreased. Site-specific analyses showed some OPS/SCS were associated with increases in local crime while most were not.

**Meaning:**

These findings suggest that with the exception of break and enters, Toronto’s OPS/SCS were associated with neutral to positive improvements in local crime trends.

## Teaser

This ecological cohort study evaluates the association between overdose prevention sites and supervised consumption sites and local crime and disorder in Toronto.

## Introduction

Canada’s largest city, Toronto, has been hit hard by the ongoing opioid crisis. Since 2016 there have been over 3251 opioid-related deaths; and in 2021 the death rate exceeded 19.4 deaths per 100 000,^1^ putting it on par with cities such as New York, Houston, San Diego, Las Vegas, and Seattle.^2^ As part of the city’s harm reduction strategy, 9 overdose prevention sites and supervised consumption sites (OPS/SCS) were implemented between 2017 and 2018. Despite evidence demonstrating the health benefits of OPS/SCS for people who use unregulated drugs,^3,4^ these sites remain controversial. Critics argue OPS/SCS may increase local crime and disorder by attracting drug-related activity such as theft, assault, open drug use, and discarded equipment.^5^

Early evidence following the implementation of Vancouver’s Insite (2003) and Sydney’s Medical Injecting Centre (2001) found no changes in police-recorded thefts or robberies, drug possession, drug dealing, open drug use, or assaults.^6,7,8^ More recently, a study examining the effects of an unsanctioned SCS in the US reported assault, burglary, larceny theft, robbery, and drug-related incidents declined in the treated neighborhood but remained the same in 2 control communities.^9^ Similarly, a study examining the effects of New York’s 2 overdose prevention sites observed no significant effects on counts of violent or property crimes in aggregate.^10^ However, the study did reveal a 30.4% (95% CI, 10.4% to 54.0%) increase in aggravated assaults, plausibly offset by decreases in simple assault (−19.7%; 95% CI, −41.5% to 10.2%) and robbery (−24.5%; 95% CI, −51.6 to 18.0).^10^ Meanwhile, a second study of New York’s sites found differential effects of the SCS owing to plausible moderating factors including a target of opportunity for petty larceny near 1 site (namely, the opening of a large retail store coinciding with when the SCS was implemented) but not the other.^11^ Finally, a study investigating the association between OPS/SCS and homicide rates (specifically, fatal stabbings and shootings) in Toronto observed no increase following the implementation of sites across the city.^12^

While many studies indicate SCS do not increase neighborhood crime and may reduce public drug use, differences in the methods used and their respective limitations may explain the variations in outcomes reported. These include short observation periods, the inability to detect meaningful differences in crime trends over time, and aggregating crimes to improve statistical power but losing nuance. Furthermore, many of the studies dominating the literature are specific to well-resourced sites and, in the case of Vancouver’s Insite, set up as a pilot to demonstrate feasibility and impact.^13^ Given the ongoing equipoise and local arguments opposed to the continued operation of Toronto’s sites, we examined the association between the city’s 9 OPS/SCS and local crime using publicly available data.

## Methods

### Study Design

For this ecological study, we used an interrupted time series study design and pooled site-specific estimates for a population-level analysis. Toronto is an ethnically and racially diverse city with approximately 2.8 million residents.^14,15^ Beginning in 2017, the majority of opioid poisoning calls were observed to be concentrated in downtown neighborhoods.^16^ In response, the Overdose Prevention Society opened the first unsanctioned OPS in Moss Park in August 2017 and within a year, 9 OPS/SCS were in operation (eTable 1 in Supplement 1). This study used publicly available data and was exempt from ethics review by McGill’s institutional review board. This study followed the Strengthening the Reporting of Observational Studies in Epidemiology (STROBE) reporting guideline for observational studies.

### Data

We restricted analysis to founded crimes that occurred between January 1, 2014, and June 30, 2025, as reported in 3 publicly available Toronto Police Service datasets: Major Crime Indicators (MCI), Bicycle Thefts, and Thefts from Motor Vehicles (see eTable 2 in Supplement 1 for definitions). The MCI included records for 5 major crimes: assaults, auto thefts, break and enters, robberies, and thefts over $5000. Across all 3 datasets, each incident record included a unique identifier, date of occurrence, and the geo-coordinates of the intersection closest to the event location. For monthly precipitation and temperature data, we used Environment Canada’s Historical Weather Data for Toronto City Centre Station.^17^

### Statistical Analysis

For our primary analysis, we conducted site-specific interrupted time series (ITS) analysis with segmented negative binomial regression to test for level and trend changes on counts of crimes within 400 m (ie, approximately a quarter mile) of the 9 OPS/SCS:

outcome_jt_ = β_0_ + β_1_ × time_t_ + β_2_ × level_j_ + β_3_ × trend_jt_ + β_4_ × CERB + β_5_ × mean cooling days + β_6_ × mean heating days + β_7_ × total precipitation + ε_jt_

For intervention j at time t, where β_1_ is the underlying trend in the outcome across time, β_2_ captures the level effect immediately after implementation, and β_3_ represents the trend effect following the implementation of the local OPS/SCS relative to the underlying trend. We also controlled for the suite of temporary social security and financial aid programs provided by the federal government in response to the economic impacts of the COVID-19 pandemic, otherwise known as Canada Emergency Response Benefits (CERB, β_4_), and monthly weather using a combination of cooling, heating, and precipitation measures (β_5_ through β_7_). We used Newey-West SEs with a lag of 3 to account for data heteroscedasticity and residual autocorrelation.

To avoid double counting events that occurred within the radii of observation of 2 sites (eg, Fred Victor site opened within 400 m of Moss Park OPS) we conducted multiple analyses. For the main analysis, we assigned events that occurred outside the first site’s radius to the new site. For sensitivity analysis, we randomly assigned the event to either site. For additional sensitivity analysis, we examined the association between OPS/SCS and crime within 100 m and 200 m of each site and restricted the observation period to 24 months before and after each site’s implementation instead of using the entire observation period.

We set the month of implementation as time 0 and reported monthly sums of outcomes. For population-level estimates, we pooled site-specific ITS outcomes and reported random effect estimates owing to the varying between-site heterogeneity. A 2-sided *P *value less than .05 was considered significant. All data preparation and analyses were conducted using R version 4.3.1 in R studio (R Project for Statistical Computing), with packages geosphere, AICcmodavg, tidyverse, sf, Synth, crsuggest, cancensus, foreign, tsModel, lmtest, Epi, splines, vcd, sandwich, reshape2, SCtools, janitor, gdata, rlang, tidyselect, car, and nlme.

## Results

After removing duplicates, errors, and incidents that occurred outside city boundaries, 196 689 assaults, 64 179 auto thefts, 77 669 break and enters, 29 118 robberies, 14 424 thefts over $5000, 31 553 bicycle thefts, and 99 577 thefts from motor vehicles occurred within Toronto during the observation period. Of these, approximately 24 782 assaults (12.6%), 2054 auto thefts (3.2%), 7456 break and enters (9.6%), 4572 robberies (15.7%), 1254 thefts over $5000 (8.7%), 6058 bicycle thefts (19.2%), and 8862 thefts from motor vehicles (8.9%) occurred within 400 m of the OPS/SCS locations.

**Figure 1.**
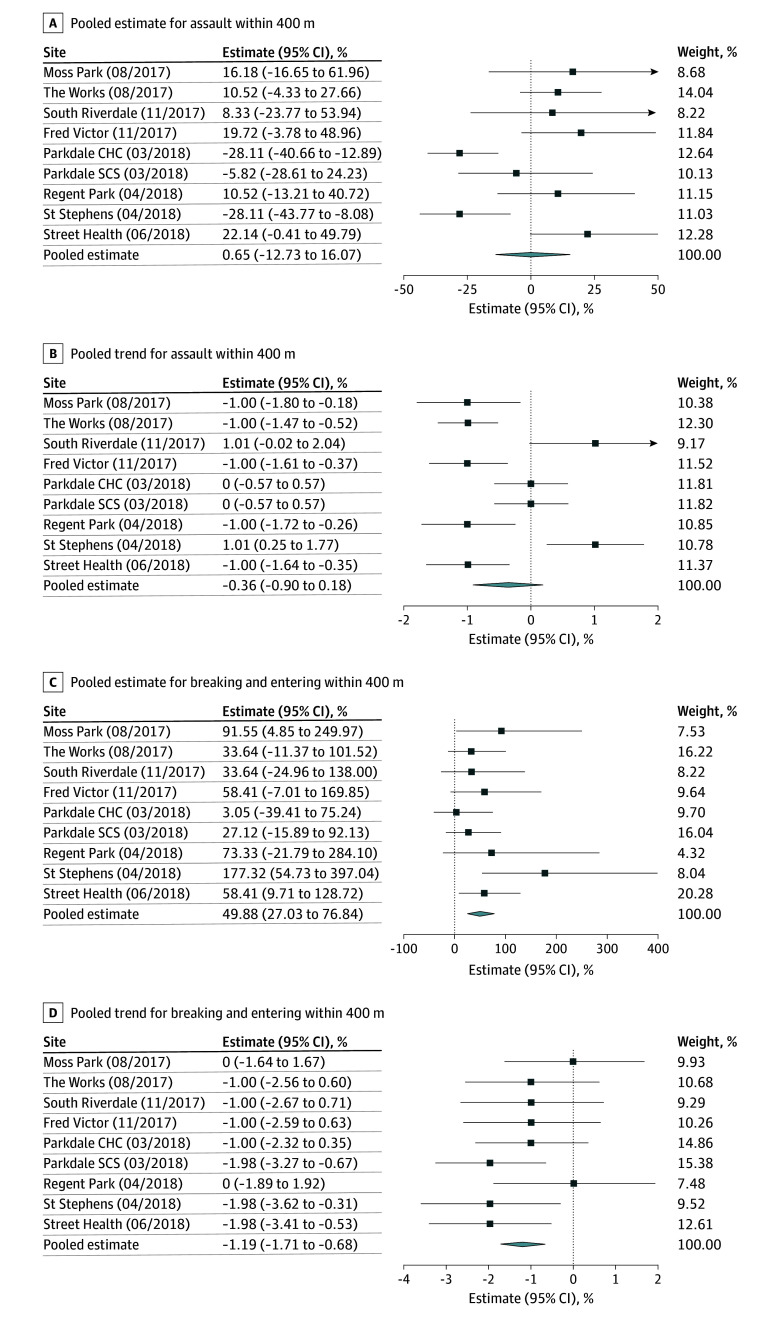
Percentage Changes in Level and Trend in Incidence of Assaults and Break and Enters Within 400 m of Overdose Prevention Sites and Supervised Consumption Sites, Per Site and Pooled CHC indicates community health center; SCS, supervised consumption site.

**Figure 2.**
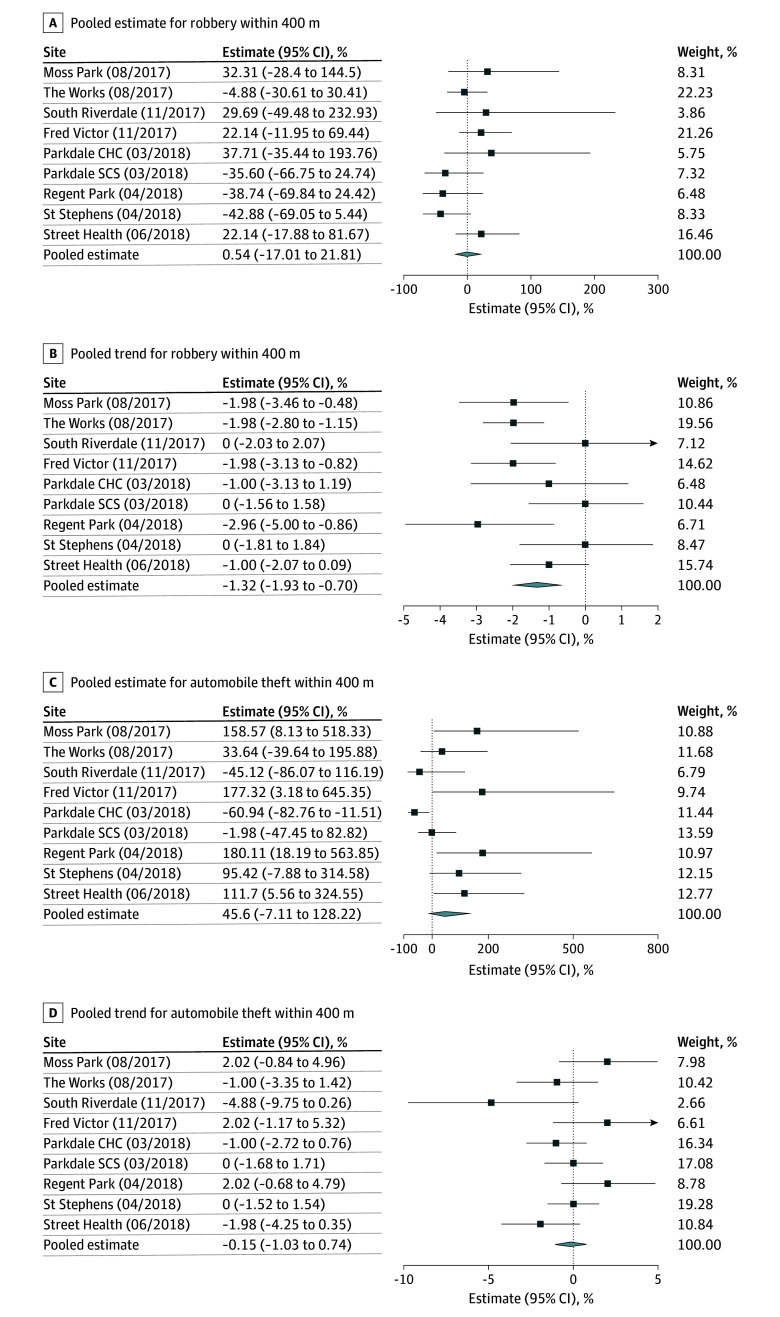
Percentage Changes in Level and Trend in Incidence of Robbery and Auto Theft Within 400 m of Overdose Prevention Sites and Supervised Consumption Sites, Per Site and Pooled CHC indicates community health center; SCS, supervised consumption site.

**Figure 3.**
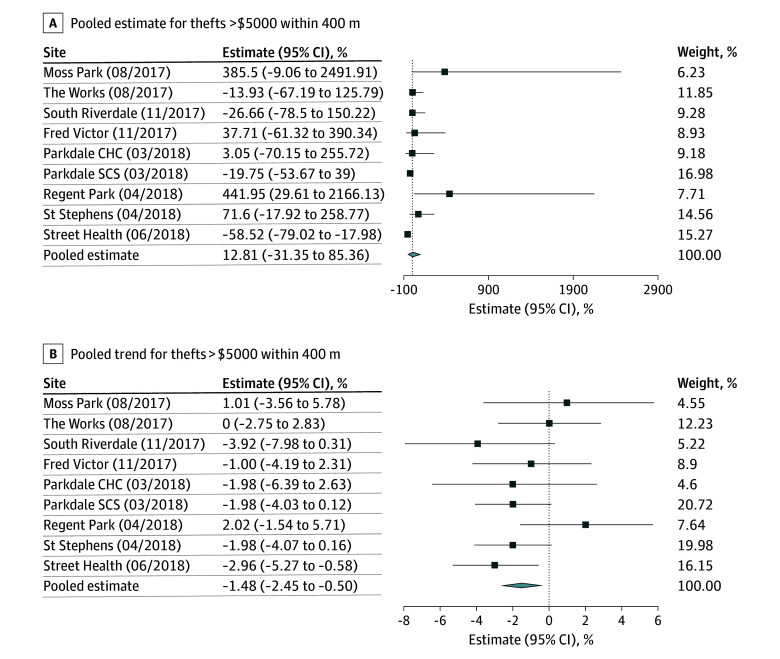
Percentage Changes in Level and Trend in Incidence of Thefts Over $5000 Within 400 m of Overdose Prevention Sites and Supervised Consumption Sites, Per Site and Pooled CHC indicates community health center; SCS, supervised consumption site.

**Figure 4.**
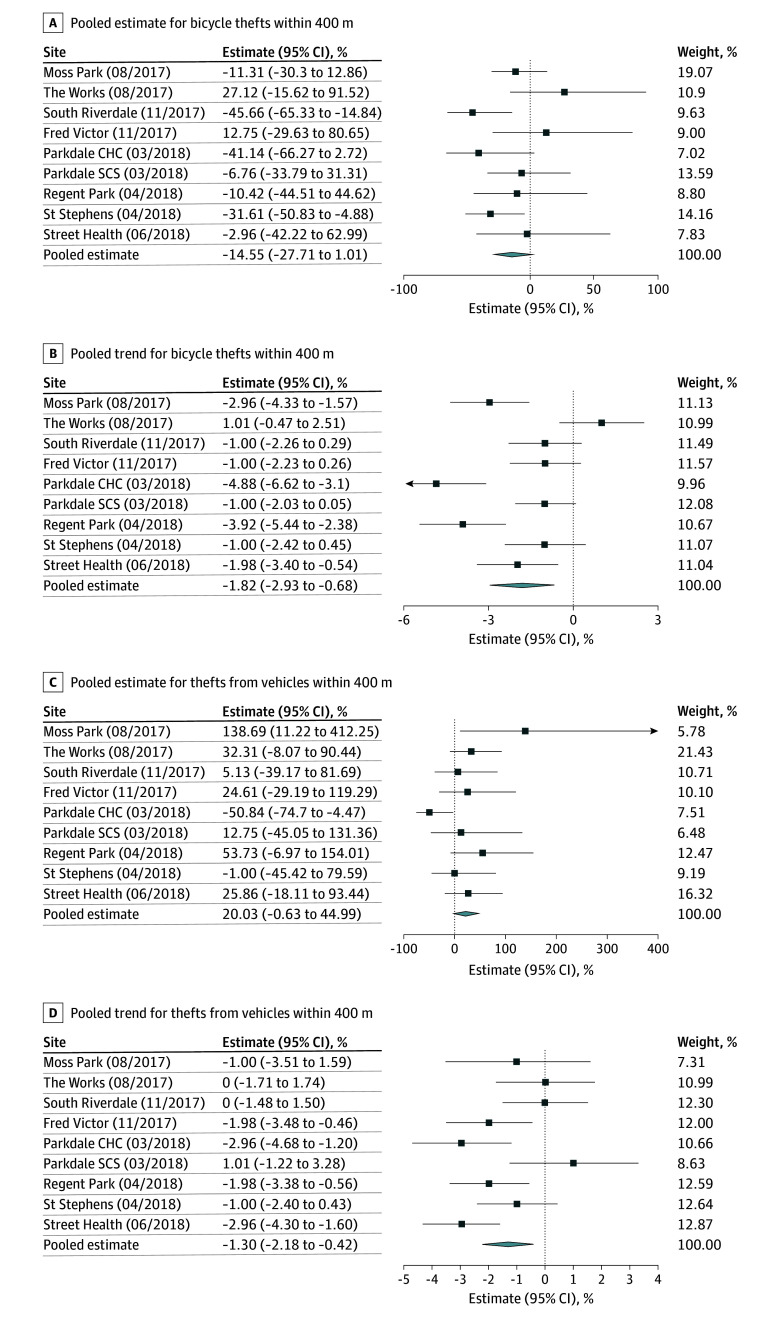
Percentage Changes in Level and Trend in Incidence of Bicycle Thefts and Thefts From Motor Vehicles Within 400 m of Overdose Prevention Sites and Supervised Consumption Sites, Per Site and Pooled CHC indicates community health center; SCS, supervised consumption site.

Figures 1, 2, 3, and 4 summarize the results of our pooled analyses at 400 m. After controlling for CERB and monthly weather, we found the opening of OPS/SCS was associated with a level increase in break and enters (49.88%; 95% CI, 27.03% to 76.84%) immediately postimplementation. However, the month-to-month trends for break and enters declined thereafter (−1.19%; 95% CI, −1.71% to −0.68%). The opening of OPS/SCS was also associated with monthly declines in bike thefts (−1.82%; 95% CI, −2.83% to −0.68%), thefts from motor vehicles (−1.30%; 95% CI, −2.18% to −0.42%), robberies (−1.32%; 95% CI, −1.93% to −0.70%), and thefts over $5000 (−1.48%; 95% CI, −2.45% to −0.50%). Disaggregated results revealed differences in level and trend effects by OPS/SCS. For example, assaults decreased within 400 m of Parkdale Community Health Centre (level effect, −28.11%; 95% CI, −40.66% to −12.89%) and St Stephen’s (level effect, −28.11%; 95% CI, −43.77% to −8.08%). Meanwhile, break and enters increased almost 2-fold near the St Stephen’s site (level effect, 177.32%; 95% CI, 54.73% to 397.04%) and for Moss Park (level effect, 91.55%; 95% CI, 4.85% to 249.97%) but otherwise did not appear to be significantly associated with most other sites.

When we restricted analysis to within 200 m of each site, the association between OPS/SCS and break and enters persisted (level effect, 64.90%; 95% CI, 11.7% to 153.45%; trend effect, −1.62%; 95% CI, −2.36% to −0.87%); as did the decline in monthly trends for thefts from motor vehicles (−1.87%; 95% CI, −3.04% to −0.69%) and robberies (−1.57%; 95% CI, −2.42% to −0.70%) (eFigures 1-4 in Supplement 1). Analysis within 100 m of each site did not detect an association between OPS/SCS and any outcomes overall, although site specific effects remained (eFigures 5-7 in Supplement 1). Similarly, when we randomly assigned the location of crime events that occurred within proximity of 2 OPS/SCS instead of assigning it to the first site in operation (main analysis) the association between OPS/SCS and break and enters remained (level effect, 45.28%; 95% CI, 19.15% to 77.13%; trend effect, −1.12%; 95% CI, −1.66% to −0.58%) (eFigures 12-15 in Supplement 1). However, when we restricted analysis to 24 months of observation before and after implementation instead of the entire observation period (main analysis) the association between OPS/SCS and break and enters diminished (level effect, −2.87%; 95% CI, −32.9% to 40.62%; trend effect, 1.79%; 95% CI, −1.16% to 4.83) (eFigures 8-11 in Supplement 1). With the exception of trends in bike thefts, no other associations between OPS/SCS and crime were detected when analyses were restricted to within 100 m of sites or limited to 24 months of observation before and after implementation.

## Discussion

Our analysis found that the opening of OPS/SCS was not associated with changes in level or trends of incidents of grand theft or crimes against persons (ie, robbery or assault) within 400 m; and the observed increase in break and enters diminished with time. A lack of association with grand theft may reflect the absence or nondifferential presence of organized criminal activity near OPS/SCS.^18^ Grand theft relies on concerted criminal networks targeting high-value items and is less sensitive to small sociodemographic changes at the community level, such as increases in people who use unregulated drugs.^19^ Conversely, associations with break and enters suggest an increase in spontaneous crimes and may be a consequence of coupling desire with the opportunity to execute said crimes.

Our results echo others’ observations that, overall, OPS/SCS did not contribute to increases in crimes.^6,9,10,11,12,20^ To contextualize our results, we consider the plausibility of 3 alternate explanations for our findings: the honey-pot effect, increased policing, and changes in data collection.

The honey-pot effect describes the phenomenon where an intervention attracts people and/or behaviors and may explain why break and enters were associated with OPS/SCS.^5^ However, this phenomenon cannot explain why the associated level change did not persist (ie, we observed neutral to declining trends over time). One reason may be that between March 2020 and August 2021, the city engaged in a large displacement of people experiencing homelessness as part of its COVID-19 public health response. During this time, the city rented 3 large hotels (ie, Bond, Edward Village, and Novotel) to provide temporary emergency shelter, effectively moving approximately 1000 people to other neighborhoods. Meanwhile, the enforcement of stringent social distancing measures scaled down OPS/SCS services.^21^ The dispersing of clients to other neighborhoods might explain the decline in spontaneous crimes. However, when measures were lifted, crime near OPS/SCS did not rebound despite a return to prepandemic rates of service use (check not shown).^22^

Similarly, while increased police presence near OPS/SCS could explain some of the trends in spontaneous crimes observed, this presents a paradox. Previous studies noted police presence undermines the social acceptability of harm reduction interventions for people who use unregulated drugs.^23^ However, outside COVID-19 restrictions, there was no notable decline in client visits. Furthermore, as far back as 2018, police budgets grew slower and the workforce decreased.^24,25^ The Toronto Police Service’s Mental Health and Addictions strategy launched in 2019 may help reconcile the shortages in the police workforce with decreases in crime reports.^26^ We concede it is possible that by improving policing quality, crimes against persons, petty theft, and break and enters decreased with minimal effect on OPS/SCS patronage, and suggest further investigation into the relationship between policing quality and crimes in Toronto.

Finally, changes in the reporting or collecting of data may explain the decline in outcomes over time. However, in 2017, police services and Statistics Canada worked together to amend the definition of founded criminal incidents to include events for which there was no credible evidence the incident did not take place.^27^ This victim-centered revision first came into effect January 1, 2018 and studies have associated it with 4% to 12% increases in counts of assault, petty theft, and fraud in subsequent years.^28^ While it is possible the public reported fewer crimes against persons near OPS/SCS, this seems unlikely when looking at citywide reporting patterns overall.^29^ For example, across the city, break and enters rose between 2017 and 2019, inclusive, declined when COVID-19 public health measures were implemented including widespread remote work policies, and rebounded beginning in 2022. However, some OPS/SCS (See Figure 1C-D; Parkdale SCS, St Stephen’s KMOPS, and Street Health) are associated with a decline in break and enters beginning as early as 2018.

Nevertheless, like Hall et al,^11^ our analysis also found crime did increase near some OPS/SCS. The local setting and preexisting context may explain some of the differences observed. For example, the St Stephen’s OPS was implemented within the St Stephen’s Community House, a site offering services for children, youths, people experiencing homelessness and/or substance use disorders, newcomers, and seniors,^30^ and was situated in Kensington Market, a neighborhood full of small shops where opportunities to break and enter are higher than elsewhere in the city. In this setting, the influx of new clients may have enabled additional spontaneous crimes. Efforts to minimize break and enters (eg, modernizing doorways and security) by residents and business owners, as well as declines in event reporting to prevent increases to insurance payments may partially explain the downward trend observed after an initial spike in events postimplementation. Meanwhile, Moss Park’s OPS was established in a local park where no other harm reduction services were offered before the OPS’s implementation. However, there were 4 parking garages located within 400 m of the site, presenting an opportunity for increases in thefts from motor vehicles and vehicle thefts as observed postimplementation.

### Limitations and Strengths

We did not include all OPS/SCS operating during the observation period. We excluded Casey House and shelters that provided services to residents only. Casey House became the first hospital to offer supervised consumption services to inpatients in August 2021 before expanding services to outpatients in April 2022. Because of differences in clientele, scope, duration of operation, and proximity to other sites, we excluded Casey House to minimize potentially biasing our analyses. Similarly, we did not include shelters that provided overdose prevention services to their residents only, including the 3 temporary housing units implemented during COVID-19 social distancing efforts, as these are qualitatively different from OPS/SCS open to the public.

Furthermore, we were unable to account for time-varying differential changes. For example, the observation period overlapped with significant upheaval in the housing market, which may have contributed to differences in the degree of people experiencing homelessness near OPS/SCS. Owing to data availability, this remains a potential source of bias in our results.

Additionally, we did not investigate the association between the openings of OPS/SCS and public drug use, needle and syringe debris, graffiti, or public defecation, concerns repeatedly mentioned by opponents of OPS/SCS.^31^ While we explored the possibility of including 311 calls in our study, we determined the quality of data insufficient and prone to reporting bias.

To our knowledge, this was the first study to examine the association of multiple OPS/SCS implemented across a large metropolitan area for a variety of crimes. Owing to the number of sites and time elapsed since their implementation, we were sufficiently powered to explore the association between OPS/SCS and a variety of measures separately, over time, and by proximity to sites. By pooling effect estimates, we reduced the potential for unmeasured contemporaneous interventions biasing our results. We limited the risk of overfitting our models and achieving spurious pretreatment fit by restricting analysis to 3 years.^32^ Further, by using exclusively open access data and providing our code, we minimized the possibility for data preparation or analytic errors and increased the transparency of our work. Lastly, we considered other explanations for our observed effects and found the available evidence did not support these alternate explanations.

## Conclusion

Our analysis revealed the implementation of Toronto’s OPS/SCS had nuanced site-specific associations with crime. Overall, the association between OPS/SCS and crimes ranging from assault to bicycle theft were neutral to positive over time. This does not negate the increases in spontaneous crimes near some sites, but rather suggests efforts to improve crime while encouraging use of OPS/SCS are possible. Still, local communities may perceive OPS/SCS as harmful, associating them with disorder and increased drug use in public spaces.^33^ This tension needs to be addressed to ensure the long term acceptability and utility of OPS/SCS. Efforts to establish relationships with local community stakeholders and to work collectively through challenges can build goodwill and trust between OPS/SCS operators, clients, and the public. Meanwhile, efforts to dismiss or negate the public’s concerns can undermine the public health intervention.

## Notes

Supplement 1. eTable 1. OPS/SCS Location, Neighborhood and Dates of Implementation eTable 2. Definitions of Each Outcome Included eFigure 1. Percentage Changes in Level (Left) and Trend (Right) in Incidence of Assaults (Top) and Break and Enters (Bottom) Within 200m of OPS/SCS, Per Site and Pooled eFigure 2. Percentage Changes in Level (Left) and Trend (Right) in Incidence of Robbery (Top) and Auto Theft (Bottom) Within 200m of OPS/SCS, Per Site and Pooled eFigure 3. Percentage Changes in Level (Left) and Trend (Right) in Incidence of Thefts Over $5000 Within 200m of OPS/SCS, Per Site and Pooled eFigure 4. Percentage Changes in Level (Left) and Trend (Right) in Incidence of Bicycle Thefts (Top) and Thefts From Motor Vehicles (Bottom) Within 200m of OPS/SCS, Per Site and Pooled eFigure 5. Percentage Changes in Level (Left) and Trend (Right) in Incidence of Assaults (Top) and Break and Enters (Bottom) Within 100m of OPS/SCS, Per Site and Pooled eFigure 6. Percentage Changes in Level (Left) and Trend (Right) in Incidence of Robbery (Top) and Auto Theft (Bottom) Within 100m of OPS/SCS, Per Site and Pooled eFigure 7. Percentage Changes in Level (Left) and Trend (Right) in Incidence of Bicycle Thefts (Top) and Thefts From Motor Vehicles (Bottom) Within 100m of OPS/SCS, Per Site and Pooled eFigure 8. Percentage Changes in Level (Left) and Trend (Right) in Incidence of Assaults (Top) and Break and Enters (Bottom) Within 400 m of OPS/SCS, Per Site and Pooled, 24 Months Preimplementation and Postimplementation eFigure 9. Percentage Changes in Level (Left) and Trend (Right) in Incidence of Robbery (Top) and Auto Theft (Bottom) Within 400 m of OPS/SCS, Per Site and Pooled, 24 Months Preimplementation and Postimplementation eFigure 10. Percentage Changes in Level (Left) and Trend (Right) in Incidence of Thefts Over $5000 Within 400 m of OPS/SCS, Per Site and Pooled, 24 Months Preimplementation and Postimplementation eFigure 11. Percentage Changes in Level (Left) and Trend (Right) in Incidence of Bicycle Thefts (Top) and Thefts From Motor Vehicles (Bottom) Within 400 m of OPS/SCS, Per Site and Pooled, 24 Months Preimplementation and Postimplementation eFigure 12. Percentage Changes in Level (Left) and Trend (Right) in Incidence of Assaults (Top) and Break and Enters (Bottom) Within 400 m of OPS/SCS, Per Site and Pooled, Random Assignment eFigure 13. Percentage Changes in Level (Left) and Trend (Right) in Incidence of Robbery (Top) and Auto Theft (Bottom) Within 400 m of OPS/SCS, Per Site and Pooled, Random Assignment eFigure 14. Percentage Changes in Level (Left) and Trend (Right) in Incidence of Thefts Over $5000 Within 400 m of OPS/SCS, Per Site and Pooled, Random Assignment eFigure 15. Percentage Changes in Level (Left) and Trend (Right) in Incidence of Bicycle Thefts (Top) and Thefts From Motor Vehicles (Bottom) Within 400 m of OPS/SCS, Per Site and Pooled, Random Assignment eReferences

Supplement 2. Data Sharing Statement
