# Supplementary material for: Toronto’s Supervised Consumption Sites and Local Crime
Source: JAMA Netw Open. 2025 Nov 25;8(11):e2545352. doi: 10.1001/jamanetworkopen.2025.45352 (PMC12648347; doi:10.1001/jamanetworkopen.2025.45352)
Supplement: Supplement 1. — eTable 1. OPS/SCS Location, Neighborhood and Dates of Implementation eTable 2. Definitions of Each Outcome Included eFigure 1. Percentage Changes in Level (Left) and Trend (Right) in Incidence of Assaults (Top) and Break and Enters (Bottom) Within 200m of OPS/SCS, Per Site and Pooled eFigure 2. Percentage Changes in Level (Left) and Trend (Right) in Incidence of Robbery (Top) and Auto Theft (Bottom) Within 200m of OPS/SCS, Per Site and Pooled eFigure 3. Percentage Changes in Level (Left) and Trend (Right) in Incidence of Thefts Over $5000 Within 200m of OPS/SCS, Per Site and Pooled eFigure 4. Percentage Changes in Level (Left) and Trend (Right) in Incidence of Bicycle Thefts (Top) and Thefts From Motor Vehicles (Bottom) Within 200m of OPS/SCS, Per Site and Pooled eFigure 5. Percentage Changes in Level (Left) and Trend (Right) in Incidence of Assaults (Top) and Break and Enters (Bottom) Within 100m of OPS/SCS, Per Site and Pooled eFigure 6. Percentage Changes in Level (Left) and Trend (Right) in Incidence of Robbery (Top) and Auto Theft (Bottom) Within 100m of OPS/SCS, Per Site and Pooled eFigure 7. Percentage Changes in Level (Left) and Trend (Right) in Incidence of Bicycle Thefts (Top) and Thefts From Motor Vehicles (Bottom) Within 100m of OPS/SCS, Per Site and Pooled eFigure 8. Percentage Changes in Level (Left) and Trend (Right) in Incidence of Assaults (Top) and Break and Enters (Bottom) Within 400 m of OPS/SCS, Per Site and Pooled, 24 Months Preimplementation and Postimplementation eFigure 9. Percentage Changes in Level (Left) and Trend (Right) in Incidence of Robbery (Top) and Auto Theft (Bottom) Within 400 m of OPS/SCS, Per Site and Pooled, 24 Months Preimplementation and Postimplementation eFigure 10. Percentage Changes in Level (Left) and Trend (Right) in Incidence of Thefts Over $5000 Within 400 m of OPS/SCS, Per Site and Pooled, 24 Months Preimplementation and Postimplementation eFigure 11. Percentage Changes in Level (Left) and Trend (Right) in Incidence [file jamanetwopen-e2545352-s001.pdf]

Supplemental Online Content

Panagiotoglou D, Lim J, Ingram G, et al. Toronto's supervised consumption sites and local crime. *JAMA Netw Open*. 2025;8(11):e2545352.
doi:10.1001/jamanetworkopen.2025.45352

eTable 1. OPS/SCS Location, Neighborhood and Dates of Implementation
eTable 2. Definitions of Each Outcome Included
eFigure 1. Percentage Changes in Level (Left) and Trend (Right) in Incidence of Assaults (Top) and Break and Enters (Bottom) Within 200m of OPS/SCS, Per Site and Pooled
eFigure 2. Percentage Changes in Level (Left) and Trend (Right) in Incidence of Robbery (Top) and Auto Theft (Bottom) Within 200m of OPS/SCS, Per Site and Pooled
eFigure 3. Percentage Changes in Level (Left) and Trend (Right) in Incidence of Thefts Over $5000 Within 200m of OPS/SCS, Per Site and Pooled
eFigure 4. Percentage Changes in Level (Left) and Trend (Right) in Incidence of Bicycle Thefts (Top) and Thefts From Vehicles (Bottom) Within 200m of OPS/SCS, Per Site and Pooled
eFigure 5. Percentage Changes in Level (Left) and Trend (Right) in Incidence of Assaults (Top) and Break and Enters (Bottom) Within 100m of OPS/SCS, Per Site and Pooled
eFigure 6. Percentage Changes in Level (Left) and Trend (Right) in Incidence of Robbery (Top) and Auto Theft (Bottom) Within 100m of OPS/SCS, Per Site and Pooled
eFigure 7. Percentage Changes in Level (Left) and Trend (Right) in Incidence of Bicycle Thefts (Top) and Thefts From Vehicles (Bottom) Within 100m of OPS/SCS, Per Site and Pooled
eFigure 8. Percentage Changes in Level (Left) and Trend (Right) in Incidence of Assaults (Top) and Break and Enters (Bottom) Within 400m of OPS/SCS, Per Site and Pooled, 24 Months Preimplementation and Postimplementation
eFigure 9. Percentage Changes in Level (Left) and Trend (Right) in Incidence of Robbery (Top) and Auto Theft (Bottom) Within 400m of OPS/SCS, Per Site and Pooled, 24 Months Preimplementation and Postimplementation
eFigure 10. Percentage Changes in Level (Left) and Trend (Right) in Incidence of Thefts Over $5000 Within 400m of OPS/SCS, Per Site and Pooled, 24 Months Preimplementation and Postimplementation
eFigure 11. Percentage Changes in Level (Left) and Trend (Right) in Incidence of Bicycle Thefts (Top) and Thefts From Vehicles (Bottom) Within 400m of OPS/SCS, Per Site and Pooled, 24 Months Preimplementation and Postimplementation
eFigure 12. Percentage Changes in Level (Left) and Trend (Right) in Incidence of Assaults (Top) and Break and Enters (Bottom) Within 400m of OPS/SCS, Per Site and Pooled, Random Assignment

© 2025 Panagiotoglou D et al. *JAMA Network Open*.

eFigure 13. Percentage Changes in Level (Left) and Trend (Right) in Incidence of Robbery (Top) and Auto Theft (Bottom) Within 400m of OPS/SCS, Per Site and Pooled, Random Assignment
eFigure 14. Percentage Changes in Level (Left) and Trend (Right) in Incidence of Thefts Over $5000 Within 400m of OPS/SCS, Per Site and Pooled, Random Assignment
eFigure 15. Percentage Changes in Level (Left) and Trend (Right) in Incidence of Bicycle Thefts (Top) and Thefts From Vehicles (Bottom) Within 400m of OPS/SCS, Per Site and Pooled, Random Assignment
eReferences

This supplemental material has been provided by the authors to give readers additional information about their work.

eTable 1. OPS/SCS Location, Neighborhood and Dates of Implementation

Site	Neighborhood	Geo-Coordinates (latitude, longitude)	Opened	Closed
Moss Park (OPS)	73 – Moss Park	43.65441862015198, -79.37064612294073	Aug 2017	Jan 2018*
Moss Park (OPS)	73 – Moss Park	43.65420291365607, -79.37196288883737	Jan 2018	May 2018*
Moss Park (OPS)	73 – Moss Park	43.654259892542775, -79.36950796249596	Jun 2018	
The Works (SCS)	75 – Church-Yonge	43.65666687196685, -79.37952470339941	Aug 2017	
South Riverdale Community Health Centre (SCS)	70 – South Riverdale	43.66121691864796, -79.33924024717157	Nov 2017	
Fred Victor Centre (SCS)	73 – Moss Park<br>75 – Church-Yonge	43.65354416730463, -79.37289244572942	Feb 2018	
Parkdale Queen West Community Health Centre (SCS)	85 – South Parkdale<br>86 – Roncesvalles	43.6419679071145, -79.42942348805988	Mar 2018	
Parkdale Supervised Consumption Service (OPS)	77 – Waterfront-Communities<br>78 – Kensington<br>81 – Trinity-Bellwoods<br>82 – Niagara	43.64661156407849, -79.40405931100734	Mar 2018	
Regent Park Community Health Centre (SCS)	72 – Regent Park<br>73 – Moss Park	43.65952867958244, -79.36555506291515	Apr 2018	
St. Stephens / KMOPS (OPS)	78 – Kensington	43.656560326757486, -79.4028629707574	Apr 2018	
Street Health (OPS)	74 – North St. James Town<br>75 – Church-Yonge	43.659455865049466, -79.37024379204236	Jun 2018	

*The Moss Park OPS that operated at Moss Park moved during its tenure. We aimed to capture its location as best as possible given its position could have an impact on outcomes included in the 100m – 400m analyses.

© 2025 Panagiotoglou D et al. *JAMA Network Open*.

eTable 2. Definitions of Each Outcome Included

We restricted analyses to founded crimes that occurred between 1 January 2014 and 30 June 2025; and fall under two types of offences within Canada's Criminal Code: offences against rights of property (e.g., theft) and crimes against persons. Unlike other criminal systems, there is no formal distinction between 'petty' and 'grand' theft, except by way of penalty, using $5000 as the threshold. We considered bicycle theft and theft from vehicle as petty theft, motor vehicle theft and theft over $5000 as grand theft, and assault and robbery as crimes against persons given the presence of a victim.[1] Break and enters are also offences against property but were not categorized as grand or petty theft.

Outcome	Definition
Assault	All intentional direct or indirect application and attempt of force to another person. Assault also includes attempts or threats, by act or gesture, to apply force to another person; or causes other person to believe on reasonable grounds that they have, present ability to achieve their purpose.
Auto thefts	Limited to acts of taking another person's vehicle.
Break and enters	Acts of entering places with the *intent* of committing indictable offenses (e.g., involving a dwelling house with no lawful excuse).
Robberies	Acts of taking property from a person or business using force or intimidation in the presence of a victim.
Thefts over $5000	Stealing property in excess of $5000 (excluding auto theft).
Bicycle thefts	Any occurrence where theft of bicycle occurred.
Thefts from motor vehicles	Acts of stealing property from a motor vehicle.

© 2025 Panagiotoglou D et al. *JAMA Network Open*.

eFigure 1. Percentage Changes in Level (Left) and Trend (Right) in Incidence of Assaults (Top) and Break and Enters (Bottom) Within 200m of OPS/SCS, Per Site and Pooled

Pooled Level Estimate: Assault - 200m

Site	Estimates	LCL	UCL	Weight (%)
Moss Park (08/2017)	-38.12	-59.83	-4.68	11.64
The Works (08/2017)	31	11.93	53.31	23.74
South Riverdale (11/2017)	84.04	-15.67	301.67	5
Fred Victor (11/2017)	19.72	-39.23	135.87	6.26
Parkdale CHC (03/2018)	-21.34	-57.39	45.23	7.3
Parkdale SCS (03/2018)	11.63	-26.83	70.31	11.95
Regent Park (04/2018)	7.25	-33.14	72.04	10.44
St. Stephens (04/2018)	-13.93	-47.02	39.83	10.09
Street Health (06/2018)	8.33	-25.62	57.77	13.58
**Pooled Estimate**	**4.98**	**-13.39**	**27.25**	

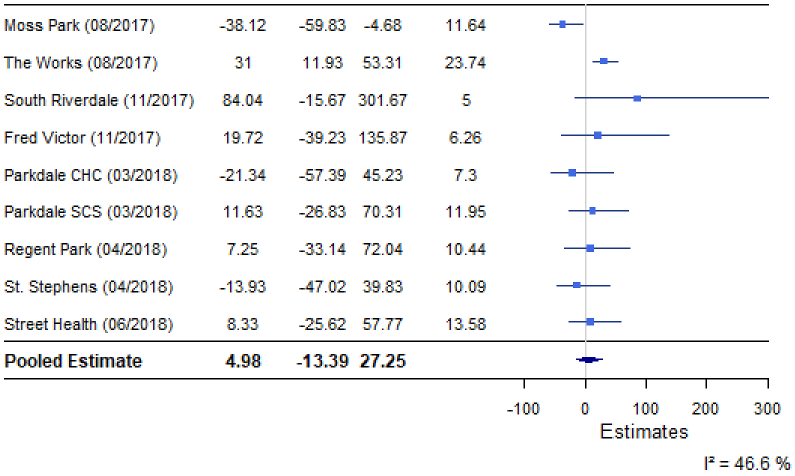

Pooled Trend Estimate: Assault - 200m

Site	Estimates	LCL	UCL	Weight (%)
Moss Park (08/2017)	-1.98	-3.34	-0.6	11.2
The Works (08/2017)	-1	-1.61	-0.38	15.35
South Riverdale (11/2017)	2.02	-0.01	4.1	8.21
Fred Victor (11/2017)	-2.96	-5.28	-0.57	6.65
Parkdale CHC (03/2018)	0	-1.83	1.86	8.95
Parkdale SCS (03/2018)	0	-1.23	1.25	12.06
Regent Park (04/2018)	0	-1.2	1.22	12.23
St. Stephens (04/2018)	1.01	-0.33	2.36	11.56
Street Health (06/2018)	-1	-1.91	-0.07	13.79
**Pooled Estimate**	**-0.43**	**-1.23**	**0.37**	

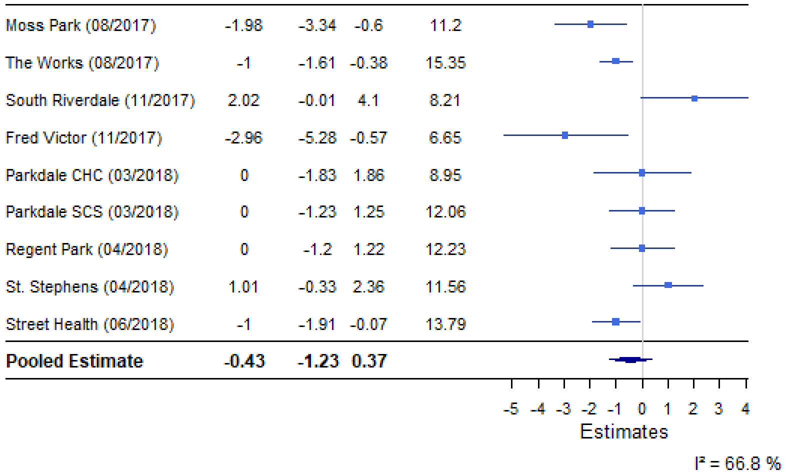

Pooled Level Estimate: B&E - 200m

Site	Estimates	LCL	UCL	Weight (%)
Moss Park (08/2017)	-1.98	-59.18	135.36	9.93
The Works (08/2017)	259.66	49.36	766.11	9.9
South Riverdale (11/2017)	-6.76	-57.19	103.08	11.11
Fred Victor (11/2017)	326.31	41.64	1183.14	7.69
Parkdale CHC (03/2018)	15.03	-58.58	219.47	8.41
Parkdale SCS (03/2018)	-12.19	-52.15	61.15	13.44
Regent Park (04/2018)	99.37	10.13	260.92	13.64
St. Stephens (04/2018)	228.71	65.61	552.42	12.34
Street Health (06/2018)	31	-28.15	138.83	13.54
**Pooled Estimate**	**64.9**	**11.7**	**143.45**	

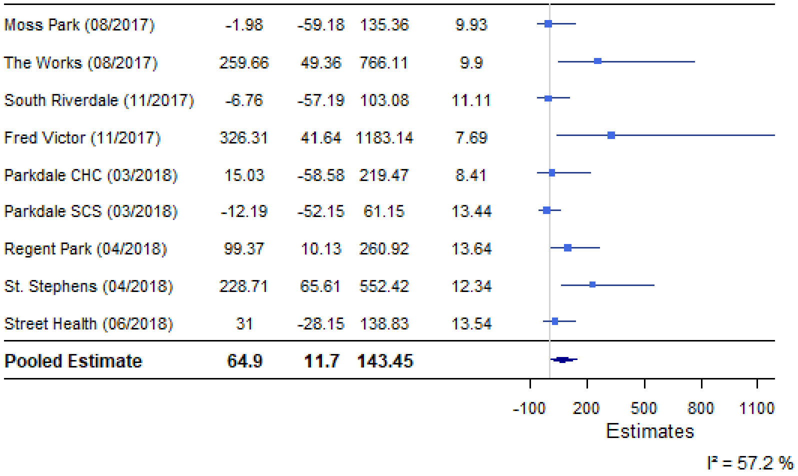

Pooled Trend Estimate: B&E - 200m

Site	Estimates	LCL	UCL	Weight (%)
Moss Park (08/2017)	-1	-3.12	1.17	12.13
The Works (08/2017)	1.01	-1.48	3.55	9.2
South Riverdale (11/2017)	-2.96	-5.22	-0.63	10.19
Fred Victor (11/2017)	-1	-4.12	2.24	5.53
Parkdale CHC (03/2018)	-1.98	-4.13	0.21	11.63
Parkdale SCS (03/2018)	-1.98	-4.04	0.12	12.68
Regent Park (04/2018)	-1	-3.4	1.47	9.41
St. Stephens (04/2018)	-1.98	-3.55	-0.39	21.9
Street Health (06/2018)	-2.96	-5.63	-0.21	7.32
**Pooled Estimate**	**-1.62**	**-2.36**	**-0.87**	

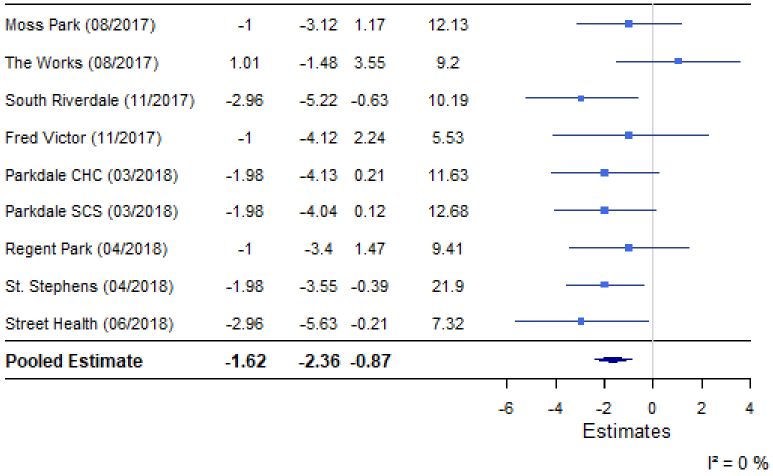

© 2025 Panagiotoglou D et al. *JAMA Network Open*.

eFigure 2. Percentage Changes in Level (Left) and Trend (Right) in Incidence of Robbery (Top) and Auto Theft (Bottom) Within 200m of OPS/SCS, Per Site and Pooled

Pooled Level Estimate: Robbery - 200m

Site	Estimates	LCL	UCL	Weight (%)
Moss Park (08/2017)	-44.57	-78.47	42.75	9.57
The Works (08/2017)	5.13	-38.73	80.37	29.38
South Riverdale (11/2017)	-50.84	-82.53	38.36	8
Fred Victor (11/2017)	13.88	-61.24	234.6	7.37
Parkdale CHC (03/2018)	-1.98	-76.67	311.86	4.15
Regent Park (04/2018)	5.13	-68.27	248.3	5.97
St. Stephens (04/2018)	5.13	-63.82	205.43	7.53
Street Health (06/2018)	-6.76	-46.35	62.04	28.03
**Pooled Estimate**	**-9.76**	**-32.65**	**20.92**	

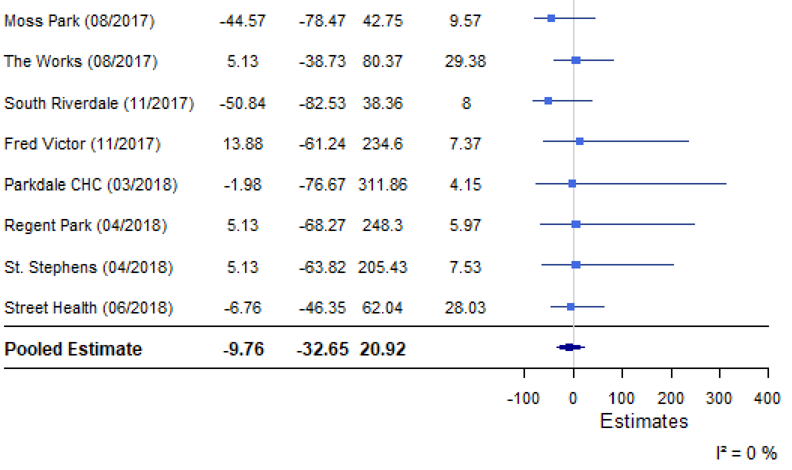

Pooled Trend Estimate: Robbery - 200m

Site	Estimates	LCL	UCL	Weight (%)
Moss Park (08/2017)	-1	-3.4	1.47	12.55
The Works (08/2017)	-1.98	-3.45	-0.49	33.36
South Riverdale (11/2017)	-1	-4.9	3.07	4.69
Fred Victor (11/2017)	-2.96	-6.75	1	4.77
Parkdale CHC (03/2018)	-2.96	-7.36	1.66	3.52
Regent Park (04/2018)	-3.92	-7.63	-0.06	4.89
St. Stephens (04/2018)	0	-3.27	3.38	6.88
Street Health (06/2018)	-1	-2.58	0.61	29.33
**Pooled Estimate**	**-1.57**	**-2.42**	**-0.7**	

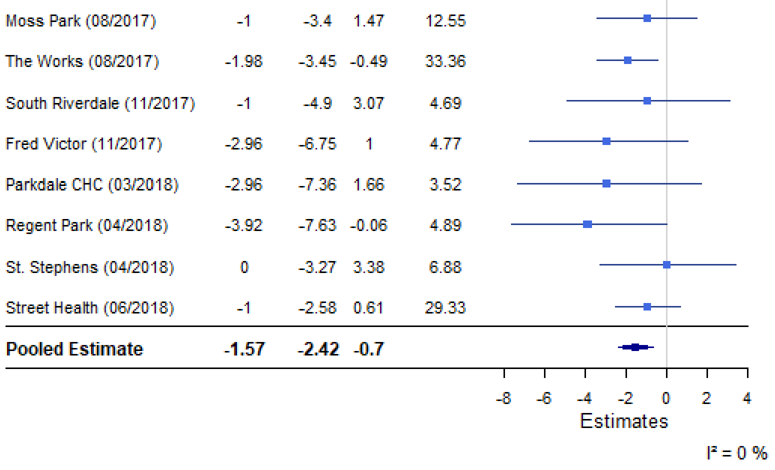

Pooled Level Estimate: Auto Theft - 200m

Site	Estimates	LCL	UCL	Weight (%)
Moss Park (08/2017)	16.18	-93.16	1872.08	3.05
The Works (08/2017)	84.04	-39.58	460.65	19.69
South Riverdale (11/2017)	-82.09	-97.4	23.17	6.57
Fred Victor (11/2017)	218.99	-40.01	1596.29	8.75
Parkdale CHC (03/2018)	-1.98	-88.8	757.85	5.19
Parkdale SCS (03/2018)	-17.3	-77.23	200.27	14.69
Regent Park (04/2018)	305.52	-11.46	1757.37	10.55
St. Stephens (04/2018)	148.43	-20.19	673.29	18.95
Street Health (06/2018)	111.7	-47.5	753.62	12.57
**Pooled Estimate**	**64.64**	**0.43**	**169.89**	

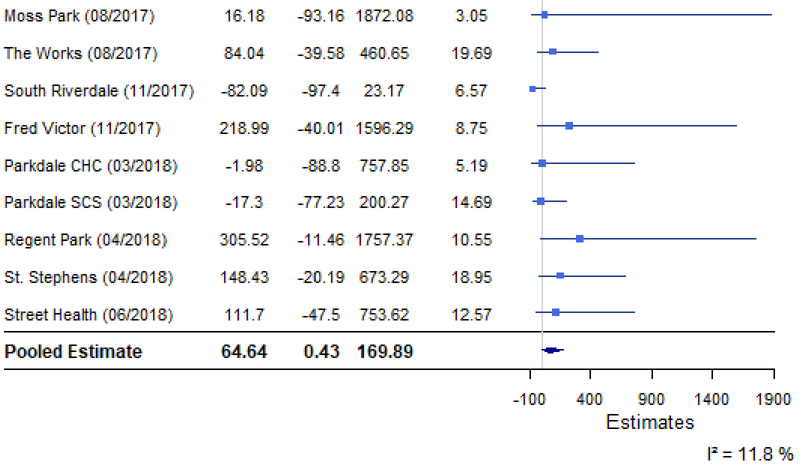

Pooled Trend Estimate: Auto Theft - 200m

Site	Estimates	LCL	UCL	Weight (%)
Moss Park (08/2017)	8.33	0.34	16.95	7.37
The Works (08/2017)	-2.96	-7.21	1.49	12.29
South Riverdale (11/2017)	-15.63	-27.39	-1.98	2.7
Fred Victor (11/2017)	-1.98	-6.38	2.62	12.09
Parkdale CHC (03/2018)	4.08	-2.11	10.66	9.44
Parkdale SCS (03/2018)	1.01	-2.07	4.17	15.04
Regent Park (04/2018)	5.13	0.99	9.44	13.2
St. Stephens (04/2018)	1.01	-2.09	4.2	14.99
Street Health (06/2018)	-3.92	-7.85	0.18	12.88
**Pooled Estimate**	**0.34**	**-2.3**	**3.05**	

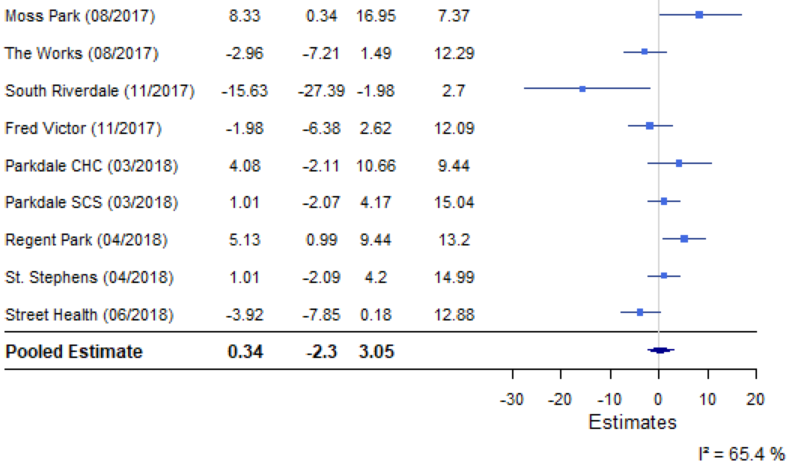

© 2025 Panagiotoglou D et al. *JAMA Network Open*.

eFigure 3. Percentage Changes in Level (Left) and Trend (Right) in Incidence of Thefts Over $5000 Within 200m of OPS/SCS, Per Site and Pooled

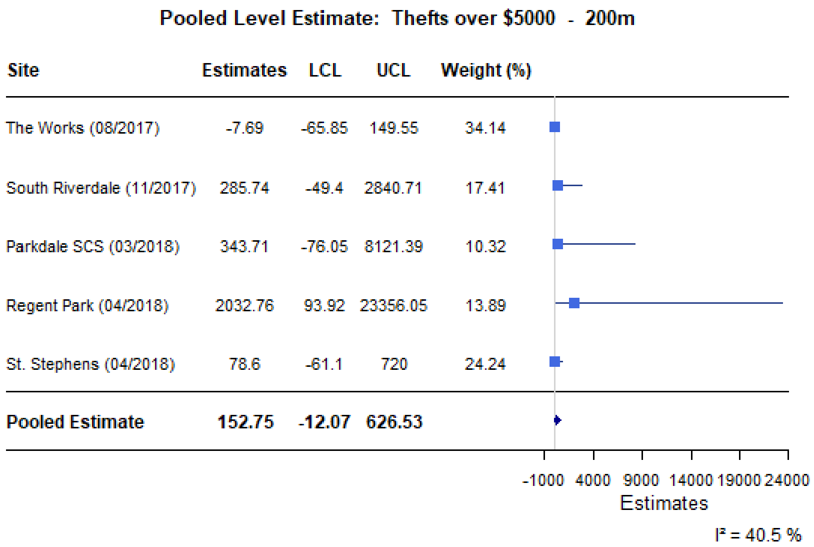

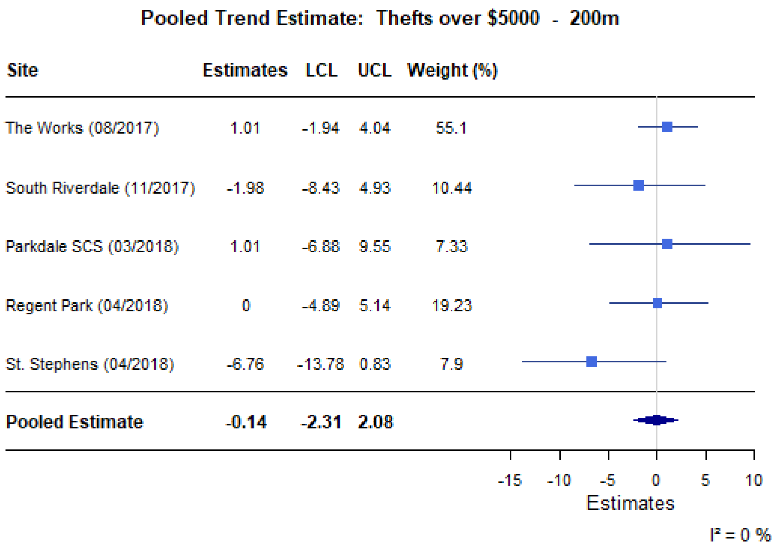

© 2025 Panagiotoglou D et al. *JAMA Network Open*.

eFigure 4. Percentage Changes in Level (Left) and Trend (Right) in Incidence of Bicycle Thefts (Top) and Thefts From Vehicles (Bottom) Within 200m of OPS/SCS, Per Site and Pooled

Pooled Level Estimate: Bicycle Thefts - 200m

Site	Estimates	LCL	UCL	Weight (%)
Moss Park (08/2017)	46.23	-27.6	195.34	12.8
The Works (08/2017)	56.83	17.94	108.54	17.12
South Riverdale (11/2017)	2.02	-50.44	110.01	12.59
Fred Victor (11/2017)	109.59	-45.42	704.93	7.1
Parkdale CHC (03/2018)	-71.35	-86.84	-37.63	11.99
Parkdale SCS (03/2018)	-35.6	-74.84	64.88	10.34
Regent Park (04/2018)	37.71	-28.08	163.7	13.39
St. Stephens (04/2018)	-50.34	-70.91	-15.23	14.67
**Pooled Estimate**	**-7.18**	**-41.28**	**46.73**	

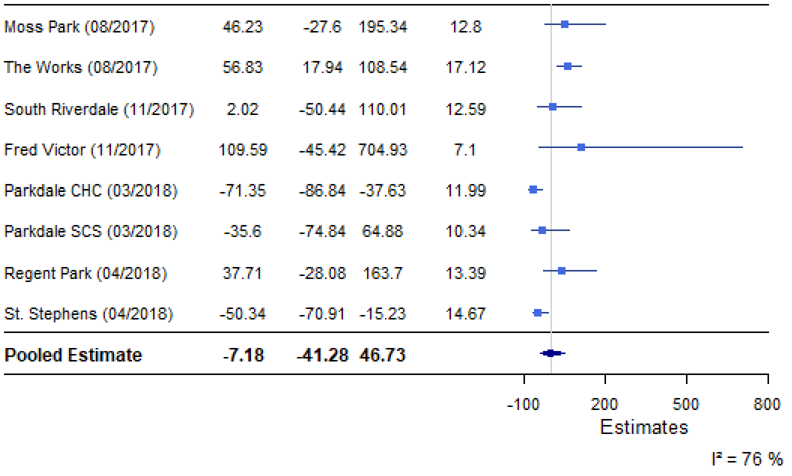

Pooled Trend Estimate: Bicycle Thefts - 200m

Site	Estimates	LCL	UCL	Weight (%)
Moss Park (08/2017)	-1.98	-5.34	1.5	9.02
The Works (08/2017)	1.01	0	2.02	18.19
South Riverdale (11/2017)	0	-2.21	2.26	13.37
Fred Victor (11/2017)	0	-3.46	3.59	8.93
Parkdale CHC (03/2018)	-2.96	-5.66	-0.18	11.14
Parkdale SCS (03/2018)	0	-2.5	2.56	12.19
Regent Park (04/2018)	-3.92	-6.4	-1.38	11.89
St. Stephens (04/2018)	-2.96	-4.65	-1.23	15.27
**Pooled Estimate**	**-1.26**	**-2.64**	**0.15**	

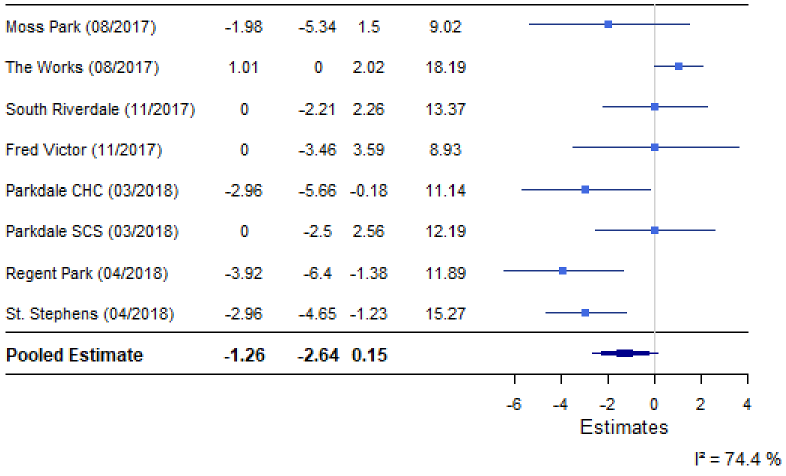

Pooled Level Estimate: Thefts from Vehicles - 200m

Site	Estimates	LCL	UCL	Weight (%)
Moss Park (08/2017)	111.7	-27.14	515.1	8.61
The Works (08/2017)	-12.19	-46.98	45.43	18.36
South Riverdale (11/2017)	76.83	-52.13	553.14	6.42
Fred Victor (11/2017)	145.96	-11.94	586.97	9.06
Parkdale CHC (03/2018)	6.18	-60.71	186.95	9.46
Parkdale SCS (03/2018)	-49.34	-85.64	78.77	6.77
Regent Park (04/2018)	156	35.82	382.52	15.45
St. Stephens (04/2018)	-32.97	-70.42	51.92	11.99
Street Health (06/2018)	10.52	-45.79	125.3	13.87
**Pooled Estimate**	**25.82**	**-13.83**	**83.71**	

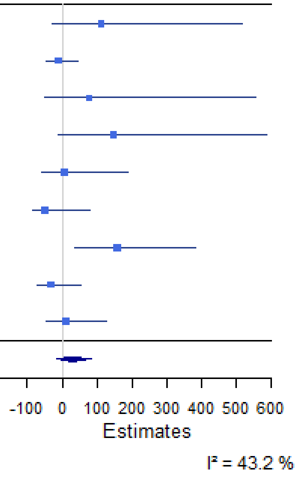

Pooled Trend Estimate: Thefts from Vehicles - 200m

Site	Estimates	LCL	UCL	Weight (%)
Moss Park (08/2017)	-1	-4.81	2.98	6.83
The Works (08/2017)	-2.96	-4.4	-1.49	18.7
South Riverdale (11/2017)	2.02	-1.85	6.04	7
Fred Victor (11/2017)	-2.96	-6.04	0.24	8.99
Parkdale CHC (03/2018)	-2.96	-6.34	0.55	7.92
Parkdale SCS (03/2018)	2.02	-1.07	5.2	9.62
Regent Park (04/2018)	-2.96	-4.94	-0.93	14.78
St. Stephens (04/2018)	-1.98	-4.06	0.15	14.25
Street Health (06/2018)	-2.96	-5.41	-0.44	11.93
**Pooled Estimate**	**-1.87**	**-3.04**	**-0.69**	

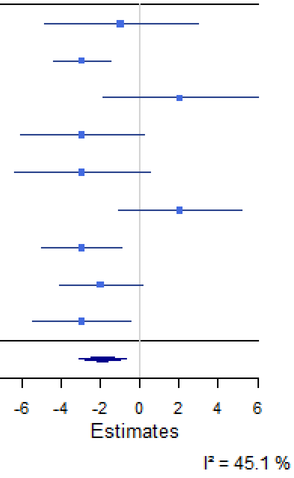

© 2025 Panagiotoglou D et al. *JAMA Network Open*.

eFigure 5. Percentage Changes in Level (Left) and Trend (Right) in Incidence of Assaults (Top) and Break and Enters (Bottom) Within 100m of OPS/SCS, Per Site and Pooled

Pooled Level Estimate: Assault - 100m

Site	Estimates	LCL	UCL	Weight (%)
The Works (08/2017)	245.56	65.61	621.05	13.85
South Riverdale (11/2017)	17.35	-63.47	276.95	7.89
Fred Victor (11/2017)	-25.92	-62.76	47.37	14.78
Parkdale CHC (03/2018)	91.55	-32.67	444.94	9.18
Parkdale SCS (03/2018)	2.02	-36.79	64.66	19.44
Regent Park (04/2018)	18.53	-34.88	115.73	16.65
St. Stephens (04/2018)	-13.06	-48.86	47.8	18.2
**Pooled Estimate**	**22.93**	**-16.58**	**81.17**	

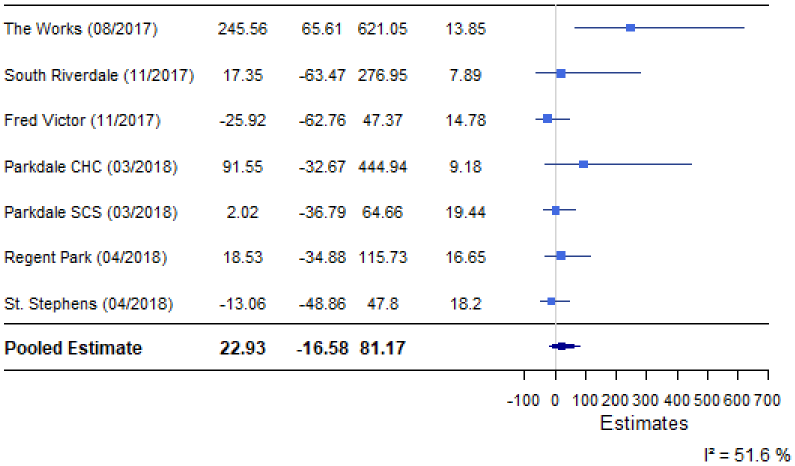

Pooled Trend Estimate: Assault - 100m

Site	Estimates	LCL	UCL	Weight (%)
The Works (08/2017)	-1.98	-4.14	0.23	8.11
South Riverdale (11/2017)	0	-3.2	3.3	3.82
Fred Victor (11/2017)	-4.88	-7.91	-1.74	3.84
Parkdale CHC (03/2018)	2.02	-1.47	5.63	3.34
Parkdale SCS (03/2018)	0	-1.11	1.12	32.24
Regent Park (04/2018)	0	-1.18	1.2	28.48
St. Stephens (04/2018)	0	-1.4	1.42	20.18
**Pooled Estimate**	**-0.29**	**-0.92**	**0.35**	

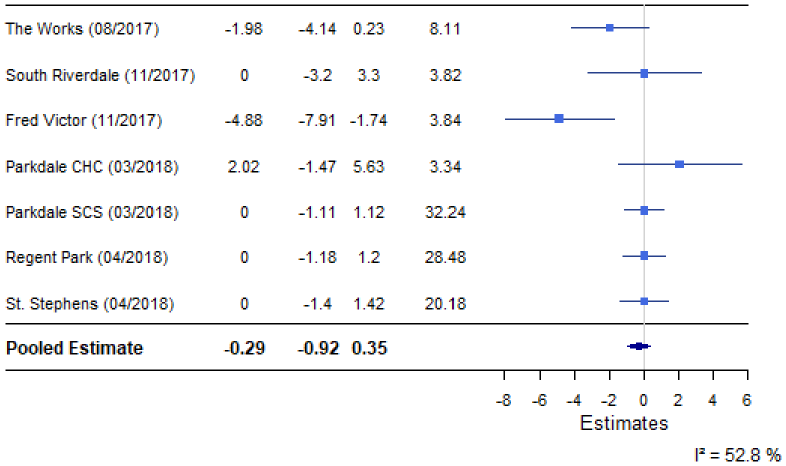

Pooled Level Estimate: B&E - 100m

Site	Estimates	LCL	UCL	Weight (%)
The Works (08/2017)	50.68	-58.07	441.46	20.39
South Riverdale (11/2017)	-28.11	-86.36	278.81	14.41
Fred Victor (11/2017)	948.56	3.91	10481.44	8.62
Parkdale SCS (03/2018)	1.01	-53.91	121.34	32.69
St. Stephens (04/2018)	289.62	27.97	1086.22	23.88
**Pooled Estimate**	**76.23**	**-16.24**	**270.78**	

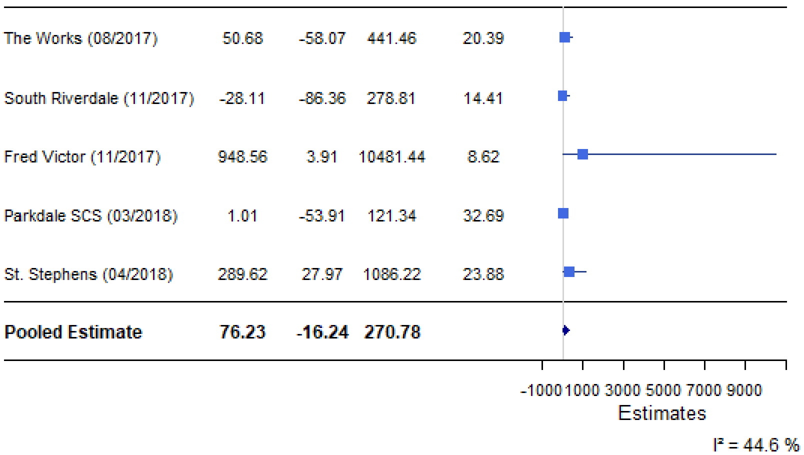

Pooled Trend Estimate: B&E - 100m

Site	Estimates	LCL	UCL	Weight (%)
The Works (08/2017)	0	-4.9	5.16	10.55
South Riverdale (11/2017)	-2.96	-9.49	4.05	5.49
Fred Victor (11/2017)	1.01	-4.86	7.23	7.46
Parkdale SCS (03/2018)	-1.98	-4.19	0.29	51.09
St. Stephens (04/2018)	-1	-4.15	2.26	25.42
**Pooled Estimate**	**-1.36**	**-2.95**	**0.27**	

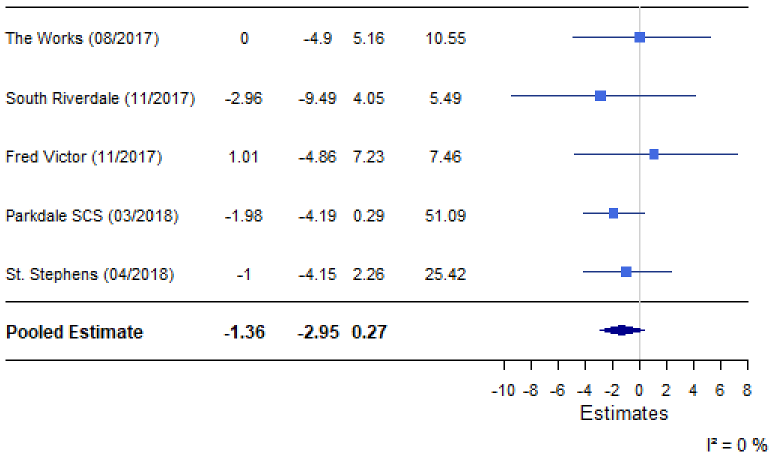

© 2025 Panagiotoglou D et al. *JAMA Network Open*.

eFigure 6. Percentage Changes in Level (Left) and Trend (Right) in Incidence of Robbery (Top) and Auto Theft (Bottom) Within 100m of OPS/SCS, Per Site and Pooled

Pooled Level Estimate: Robbery - 100m

Site	Estimates	LCL	UCL	Weight (%)
South Riverdale (11/2017)	-91.87	-98.84	-43.04	20.44
Fred Victor (11/2017)	-1.98	-64.88	173.54	28.64
Regent Park (04/2018)	182.92	-24.08	954.31	26.05
St. Stephens (04/2018)	127.05	-46.49	863.45	24.87
**Pooled Estimate**	**-4.3**	**-76.37**	**287.52**	

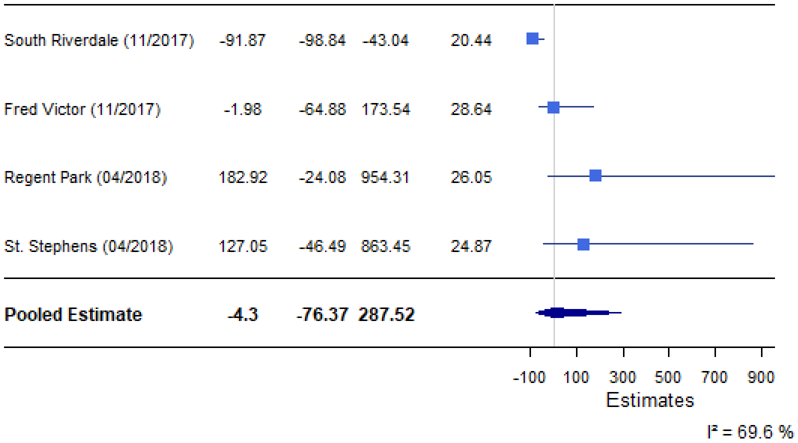

Pooled Trend Estimate: Robbery - 100m

Site	Estimates	LCL	UCL	Weight (%)
South Riverdale (11/2017)	-4.88	-13.54	4.65	12.34
Fred Victor (11/2017)	-4.88	-8.59	-1.01	29.84
Regent Park (04/2018)	-4.88	-9.08	-0.48	27.48
St. Stephens (04/2018)	2.02	-1.86	6.05	30.34
**Pooled Estimate**	**-2.84**	**-6.63**	**1.11**	

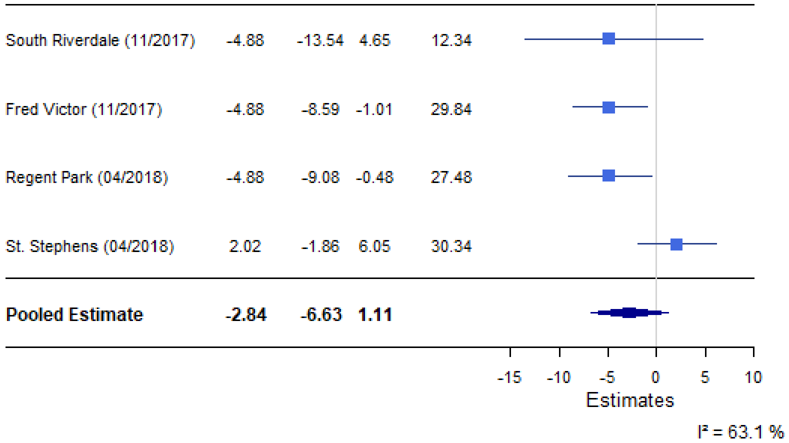

Pooled Level Estimate: Auto Theft - 100m

Site	Estimates	LCL	UCL	Weight (%)
South Riverdale (11/2017)	-98.98	-99.97	-64.06	31.01
Fred Victor (11/2017)	767.11	-76.58	32001.53	30.77
Parkdale SCS (03/2018)	-83.8	-98.25	50.16	38.22
**Pooled Estimate**	**-76.64**	**-99.34**	**730.66**	

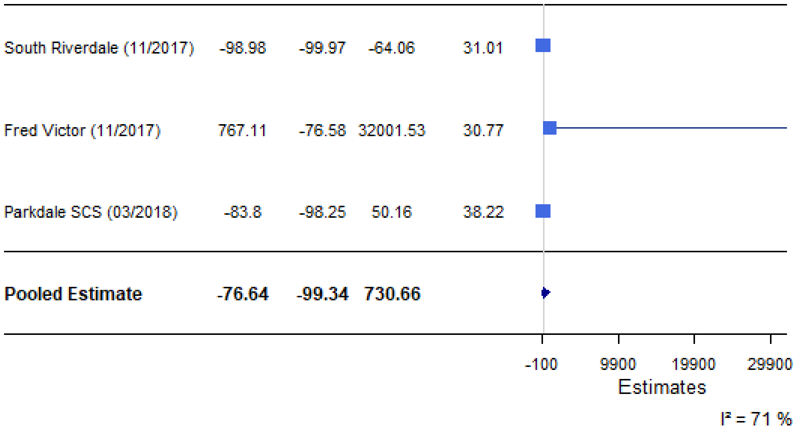

Pooled Trend Estimate: Auto Theft - 100m

Site	Estimates	LCL	UCL	Weight (%)
South Riverdale (11/2017)	-29.53	-42.73	-13.29	28.21
Fred Victor (11/2017)	3.05	-5.8	12.72	35.12
Parkdale SCS (03/2018)	2.02	-2.47	6.72	36.67
**Pooled Estimate**	**-7.77**	**-26.27**	**15.38**	

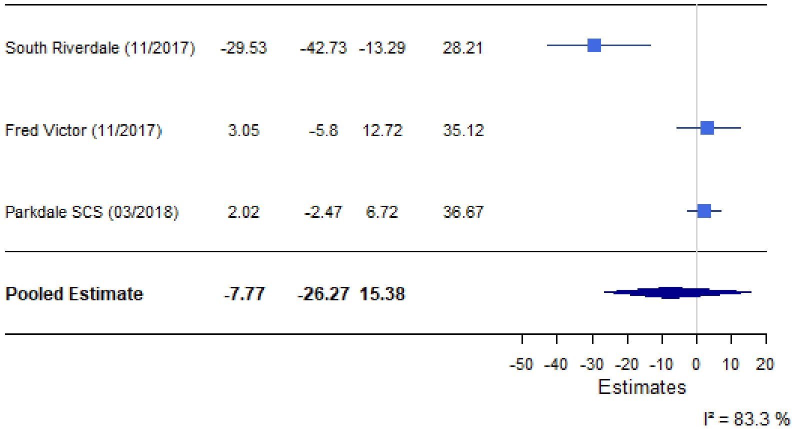

© 2025 Panagiotoglou D et al. *JAMA Network Open*.

eFigure 7. Percentage Changes in Level (Left) and Trend (Right) in Incidence of Bicycle Thefts (Top) and Thefts From Motor Vehicles (Bottom) Within 100m of OPS/SCS, Per Site and Pooled

Pooled Level Estimate: Bicycle Thefts - 100m

Site	Estimates	LCL	UCL	Weight (%)
The Works (08/2017)	-12.19	-80.52	295.78	19.22
Parkdale CHC (03/2018)	-97.16	-99.69	-73.49	13.12
Parkdale SCS (03/2018)	-24.42	-75.47	132.85	23.2
Regent Park (04/2018)	37.71	-75.06	660.43	17.29
St. Stephens (04/2018)	37.71	-34.86	191.12	27.17
**Pooled Estimate**	**-33.94**	**-77.09**	**90.48**	

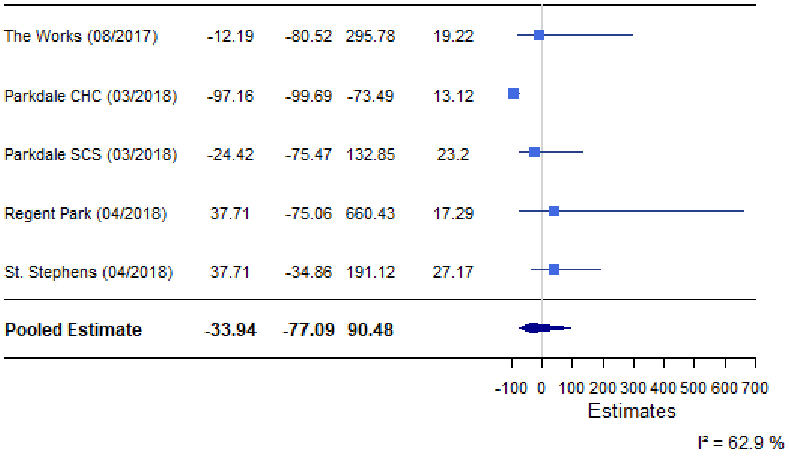

Pooled Trend Estimate: Bicycle Thefts - 100m

Site	Estimates	LCL	UCL	Weight (%)
The Works (08/2017)	0	-4.08	4.26	16.72
Parkdale CHC (03/2018)	-1.98	-6.57	2.84	12.6
Parkdale SCS (03/2018)	1.01	-1.94	4.04	33.16
Regent Park (04/2018)	-1.98	-6.57	2.83	12.65
St. Stephens (04/2018)	0	-3.36	3.48	24.88
**Pooled Estimate**	**-0.17**	**-1.86**	**1.54**	

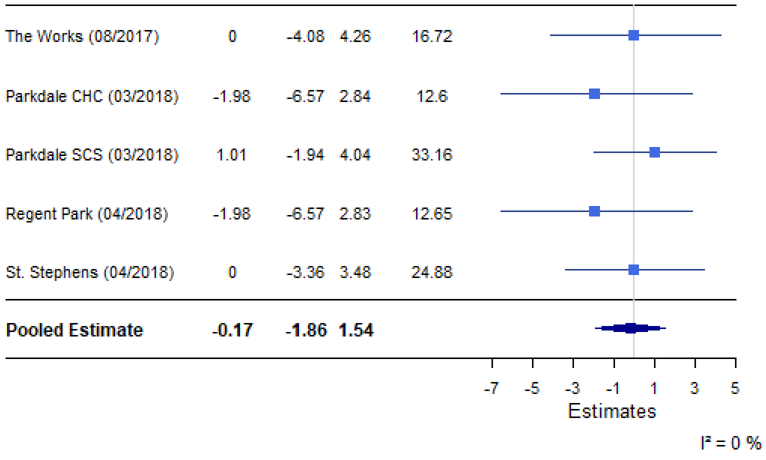

Pooled Level Estimate: Thefts from Vehicles - 100m

Site	Estimates	LCL	UCL	Weight (%)
The Works (08/2017)	-35.6	-81.96	129.97	16.76
Fred Victor (11/2017)	-6.76	-81.38	366.86	10.46
Parkdale CHC (03/2018)	69.89	-56.1	557.49	14.82
Regent Park (04/2018)	131.64	-14.16	525.04	27.55
St. Stephens (04/2018)	68.2	-34.61	332.64	30.41
**Pooled Estimate**	**47.26**	**-12.54**	**147.95**	

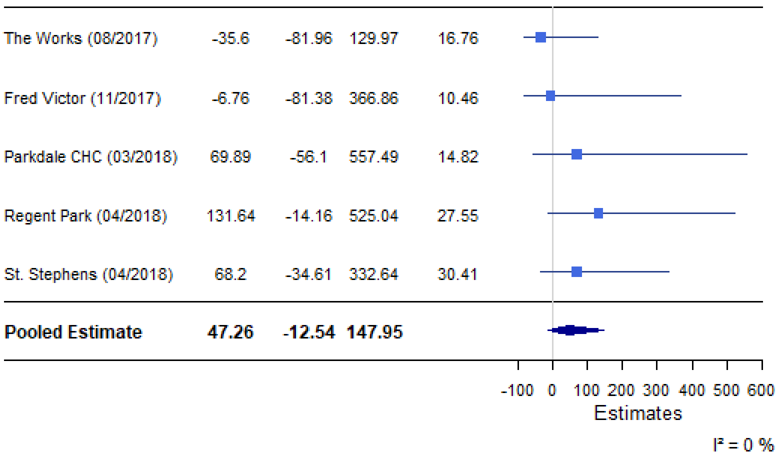

Pooled Trend Estimate: Thefts from Vehicles - 100m

Site	Estimates	LCL	UCL	Weight (%)
The Works (08/2017)	0	-3.04	3.13	29.69
Fred Victor (11/2017)	1.01	-3.18	5.38	15.76
Parkdale CHC (03/2018)	-3.92	-8.63	1.03	11.2
Regent Park (04/2018)	-4.88	-10	0.54	9.23
St. Stephens (04/2018)	-1	-3.8	1.89	34.13
**Pooled Estimate**	**-1.09**	**-2.74**	**0.59**	

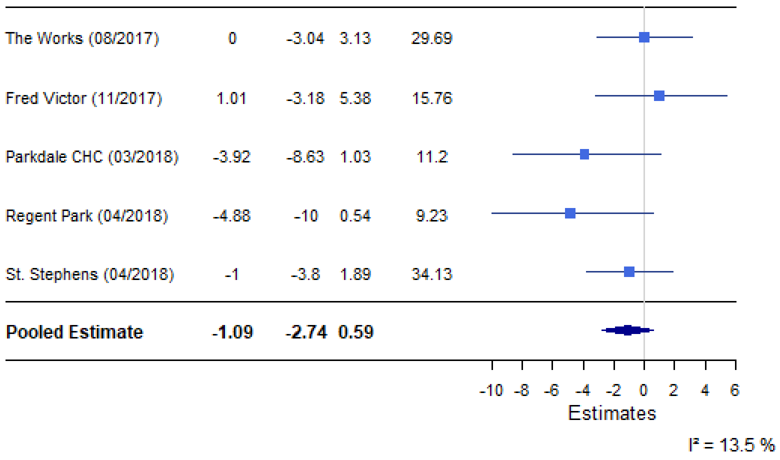

© 2025 Panagiotoglou D et al. *JAMA Network Open*.

eFigure 8. Percentage Changes in Level (Left) and Trend (Right) in Incidence of Assaults (Top) and Break and Enters (Bottom) Within 400m of OPS/SCS, Per Site and Pooled, 24 Months Preimplementation and Postimplementation

Pooled Level Estimate: Assault - 400m

Site	Estimates	LCL	UCL	Weight (%)
Moss Park (08/2017)	-17.3	-44.25	22.67	5.3
The Works (08/2017)	3.05	-10.2	18.25	43.51
South Riverdale (11/2017)	18.53	-19.09	73.65	5.65
Fred Victor (11/2017)	-7.69	-29.01	20.04	11.95
Parkdale CHC (03/2018)	-19.75	-41.37	9.84	8.37
Parkdale SCS (03/2018)	-28.82	-54.92	12.38	3.95
Regent Park (04/2018)	-2.96	-33.08	40.74	5.97
St. Stephens (04/2018)	-4.88	-32.25	33.56	7.16
Street Health (06/2018)	13.88	-17.14	56.52	8.15
**Pooled Estimate**	**-2.33**	**-10.81**	**6.95**	

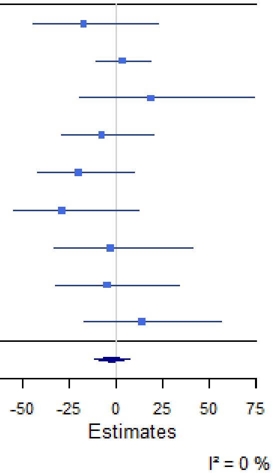

Pooled Trend Estimate: Assault - 400m

Site	Estimates	LCL	UCL	Weight (%)
Moss Park (08/2017)	3.05	0.46	5.69	9.79
The Works (08/2017)	0	-1.11	1.13	15.7
South Riverdale (11/2017)	2.02	-1.24	5.39	7.52
Fred Victor (11/2017)	-1.98	-3.85	-0.07	12.19
Parkdale CHC (03/2018)	-1	-3.24	1.3	10.69
Parkdale SCS (03/2018)	1.01	-1.89	3.99	8.52
Regent Park (04/2018)	2.02	0.16	3.92	12.56
St. Stephens (04/2018)	-1.98	-4.3	0.4	10.31
Street Health (06/2018)	-1	-2.77	0.81	12.72
**Pooled Estimate**	**0.1**	**-1.06**	**1.26**	

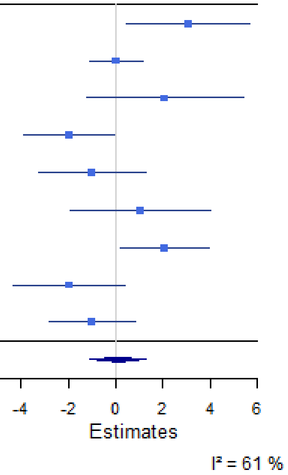

Pooled Level Estimate: B&E - 400m

Site	Estimates	LCL	UCL	Weight (%)
Moss Park (08/2017)	-29.53	-58	18.24	12.64
The Works (08/2017)	29.69	-35.55	160.99	10.5
South Riverdale (11/2017)	-26.66	-69.09	74.02	8.77
Fred Victor (11/2017)	11.63	-30.85	80.21	13.11
Parkdale CHC (03/2018)	-55.96	-78.82	-8.43	10.13
Parkdale SCS (03/2018)	-35.6	-61.05	6.5	12.82
Regent Park (04/2018)	-38.74	-82.32	112.25	5.8
St. Stephens (04/2018)	163.79	53.86	352.27	12.38
Street Health (06/2018)	41.91	-6.44	115.24	13.85
**Pooled Estimate**	**-2.87**	**-32.9**	**40.62**	

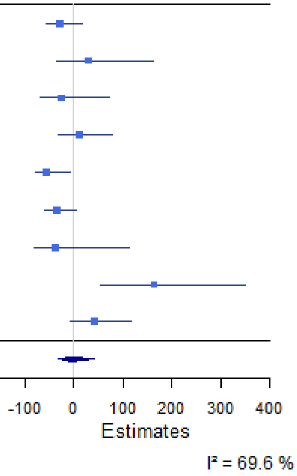

Pooled Trend Estimate: B&E - 400m

Site	Estimates	LCL	UCL	Weight (%)
Moss Park (08/2017)	10.52	6.74	14.43	11.77
The Works (08/2017)	0	-3.27	3.38	11.94
South Riverdale (11/2017)	3.05	-1.92	8.26	10.08
Fred Victor (11/2017)	-1.98	-6.81	3.1	9.95
Parkdale CHC (03/2018)	6.18	2.3	10.22	11.48
Parkdale SCS (03/2018)	3.05	0.32	5.84	12.61
Regent Park (04/2018)	-2.96	-9.37	3.92	7.99
St. Stephens (04/2018)	-3.92	-7.39	-0.33	11.55
Street Health (06/2018)	1.01	-1.64	3.72	12.63
**Pooled Estimate**	**1.79**	**-1.16**	**4.83**	

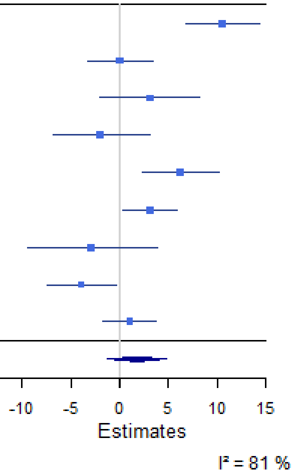

© 2025 Panagiotoglou D et al. *JAMA Network Open*.

eFigure 9. Percentage Changes in Level (Left) and Trend (Right) in Incidence of Robbery (Top) and Auto Theft (Bottom) Within 400m of OPS/SCS, Per Site and Pooled, 24 Months Preimplementation and Postimplementation

Pooled Level Estimate: Robbery - 400m

Site	Estimates	LCL	UCL	Weight (%)
Moss Park (08/2017)	-26.66	-71.36	87.81	8.02
The Works (08/2017)	19.72	-15.73	70.08	16.57
South Riverdale (11/2017)	-32.29	-76.13	92.05	7.05
Fred Victor (11/2017)	1.01	-34.26	55.19	15.26
Parkdale CHC (03/2018)	263.28	29.98	915.32	7.18
Parkdale SCS (03/2018)	-71.35	-88.09	-31.09	8.69
Regent Park (04/2018)	-28.82	-69.07	63.8	9.2
St. Stephens (04/2018)	37.71	-16.73	127.76	14.02
Street Health (06/2018)	-22.89	-53.43	27.67	14
**Pooled Estimate**	**-5.81**	**-33.23**	**32.86**	

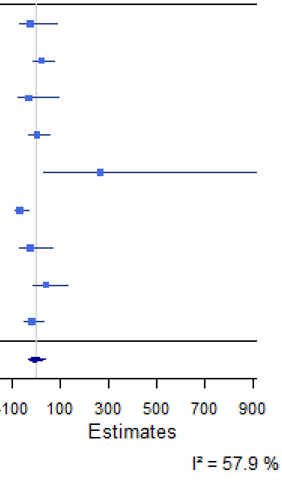

Pooled Trend Estimate: Robbery - 400m

Site	Estimates	LCL	UCL	Weight (%)
Moss Park (08/2017)	6.18	-0.1	12.87	7.61
The Works (08/2017)	-4.88	-7	-2.7	17.04
South Riverdale (11/2017)	0	-4.91	5.16	9.58
Fred Victor (11/2017)	-2.96	-7.75	2.09	9.51
Parkdale CHC (03/2018)	2.02	-4.03	8.45	7.6
Parkdale SCS (03/2018)	3.05	-2.33	8.71	8.94
Regent Park (04/2018)	-1.98	-6.55	2.81	10.13
St. Stephens (04/2018)	-1	-4.2	2.32	13.96
Street Health (06/2018)	-1	-3.66	1.74	15.63
**Pooled Estimate**	**-0.76**	**-2.82**	**1.35**	

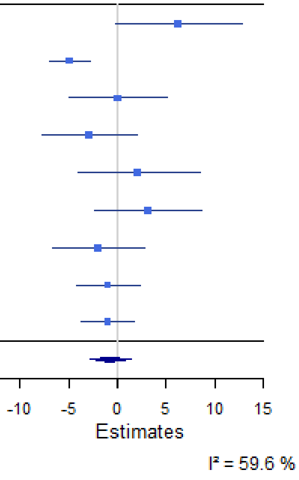

Pooled Level Estimate: Auto Theft - 400m

Site	Estimates	LCL	UCL	Weight (%)
Moss Park (08/2017)	93.48	-49.62	643.11	8.3
The Works (08/2017)	-19.75	-78.39	197.98	8.73
South Riverdale (11/2017)	169.12	-26.86	890.26	8.85
Fred Victor (11/2017)	158.57	-27.18	818.16	9.36
Parkdale CHC (03/2018)	-92.65	-98.85	-52.98	4.36
Parkdale SCS (03/2018)	27.12	-48.19	211.93	18.65
Regent Park (04/2018)	339.29	-21.32	2352.67	5.08
St. Stephens (04/2018)	68.2	-23.09	267.84	24.54
Street Health (06/2018)	60	-47.39	386.58	12.14
**Pooled Estimate**	**49.59**	**1.53**	**120.41**	

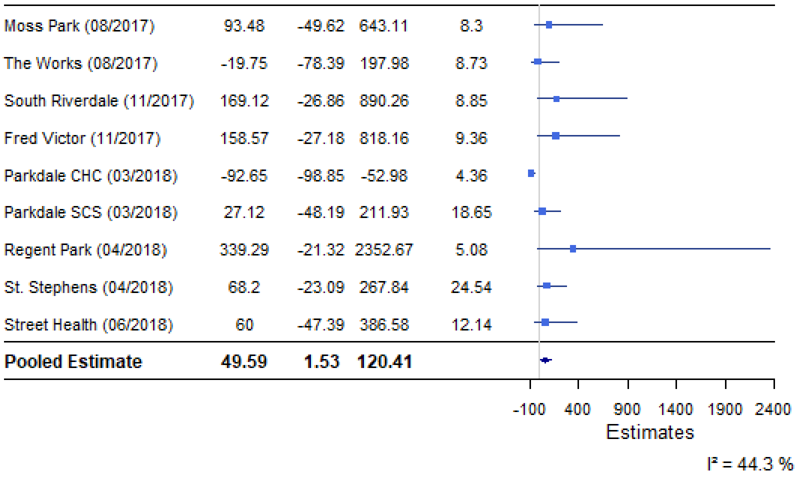

Pooled Trend Estimate: Auto Theft - 400m

Site	Estimates	LCL	UCL	Weight (%)
Moss Park (08/2017)	-1	-11.12	10.29	6.81
The Works (08/2017)	4.08	-2.69	11.32	12.14
South Riverdale (11/2017)	-15.63	-24.97	-5.14	6.03
Fred Victor (11/2017)	2.02	-4.4	8.88	12.54
Parkdale CHC (03/2018)	3.05	-7.27	14.51	7.03
Parkdale SCS (03/2018)	2.02	-2.72	6.99	16.15
Regent Park (04/2018)	3.05	-6.53	13.6	7.84
St. Stephens (04/2018)	9.42	3.59	15.57	14.59
Street Health (06/2018)	1.01	-3.38	5.58	16.88
**Pooled Estimate**	**1.91**	**-1.42**	**5.36**	

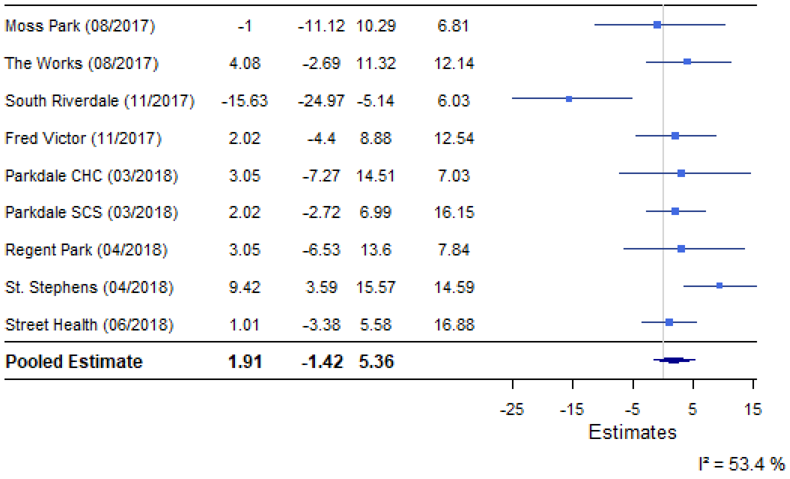

© 2025 Panagiotoglou D et al. *JAMA Network Open*.

eFigure 10. Percentage Changes in Level (Left) and Trend (Right) in Incidence of Thefts Over $5000 Within 400m of OPS/SCS, Per Site and Pooled, 24 Months Preimplementation and Postimplementation

Pooled Level Estimate: Thefts over $5000 - 400m

Site	Estimates	LCL	UCL	Weight (%)
Moss Park (08/2017)	25.86	-79.09	657.54	9.87
The Works (08/2017)	-21.34	-86.12	345.91	10.26
South Riverdale (11/2017)	-56.83	-95.39	304.07	7.47
Fred Victor (11/2017)	405.31	-54.47	5508.25	6.75
Parkdale CHC (03/2018)	-87	-95.69	-60.73	15.43
Parkdale SCS (03/2018)	-34.95	-74.84	68.16	16.95
Regent Park (04/2018)	124.79	-81.07	2569.32	6.48
St. Stephens (04/2018)	107.51	-33.92	551.67	15.05
Street Health (06/2018)	-41.14	-87.24	171.61	11.74
**Pooled Estimate**	**-21.56**	**-62.59**	**64.48**	

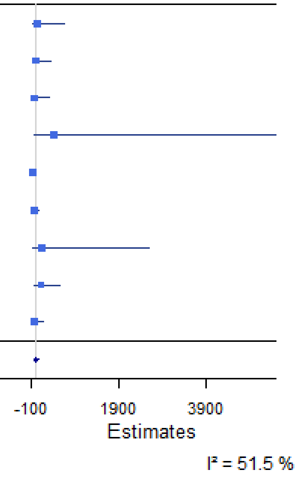

Pooled Trend Estimate: Thefts over $5000 - 400m

Site	Estimates	LCL	UCL	Weight (%)
Moss Park (08/2017)	-5.82	-20.3	11.28	6.21
The Works (08/2017)	3.05	-3.92	10.52	13.93
South Riverdale (11/2017)	1.01	-7.78	10.62	11.78
Fred Victor (11/2017)	7.25	-3.13	18.74	10.76
Parkdale CHC (03/2018)	-22.89	-32.66	-11.71	8.06
Parkdale SCS (03/2018)	-1	-6.45	4.78	15.33
Regent Park (04/2018)	-11.31	-24.99	4.87	6.18
St. Stephens (04/2018)	-1	-6.78	5.15	14.97
Street Health (06/2018)	-1	-8.7	7.36	12.78
**Pooled Estimate**	**-2.33**	**-7.16**	**2.76**	

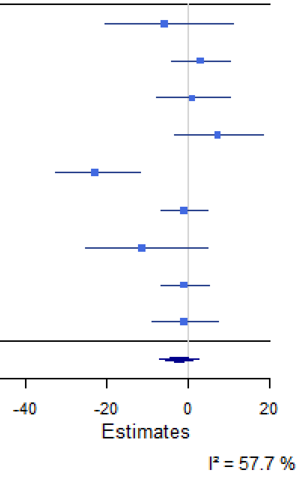

© 2025 Panagiotoglou D et al. *JAMA Network Open*.

eFigure 11. Percentage Changes in Level (Left) and Trend (Right) in Incidence of Bicycle Thefts (Top) and Thefts From Motor Vehicles (Bottom) Within 400m of OPS/SCS, Per Site and Pooled, 24 Months Preimplementation and Postimplementation

Pooled Level Estimate: Bicycle Thefts - 400m

Site	Estimates	LCL	UCL	Weight (%)
Moss Park (08/2017)	-25.92	-49.43	8.52	15.48
The Works (08/2017)	41.91	7.98	86.49	20.02
South Riverdale (11/2017)	-41.73	-73.22	26.81	6.28
Fred Victor (11/2017)	23.37	-25.29	103.72	11.57
Parkdale CHC (03/2018)	-42.88	-79.81	61.62	3.88
Parkdale SCS (03/2018)	8.33	-32.03	72.65	12.6
Regent Park (04/2018)	-22.89	-58.38	42.85	8.85
St. Stephens (04/2018)	12.75	-36.35	99.72	9.81
Street Health (06/2018)	-18.13	-50.57	35.6	11.49
**Pooled Estimate**	**-3.14**	**-22.29**	**20.73**	

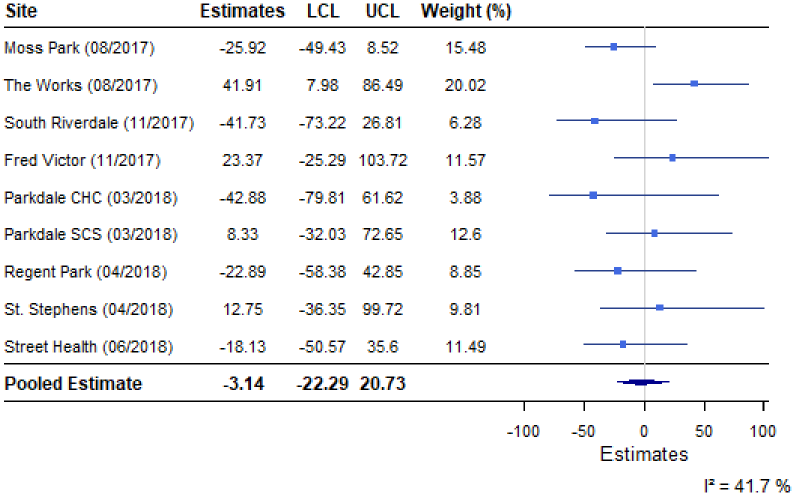

Pooled Trend Estimate: Bicycle Thefts - 400m

Site	Estimates	LCL	UCL	Weight (%)
Moss Park (08/2017)	-3.92	-6.74	-1.02	12.77
The Works (08/2017)	-1.98	-3.26	-0.68	15.15
South Riverdale (11/2017)	2.02	-1.87	6.06	11.22
Fred Victor (11/2017)	-6.76	-9.41	-4.04	12.93
Parkdale CHC (03/2018)	-3.92	-12.83	5.9	4.41
Parkdale SCS (03/2018)	0	-3.31	3.42	12.11
Regent Park (04/2018)	-8.61	-12.44	-4.6	10.55
St. Stephens (04/2018)	-3.92	-7.78	0.1	10.86
Street Health (06/2018)	1.01	-3.56	5.79	10
**Pooled Estimate**	**-2.91**	**-5.22**	**-0.55**	

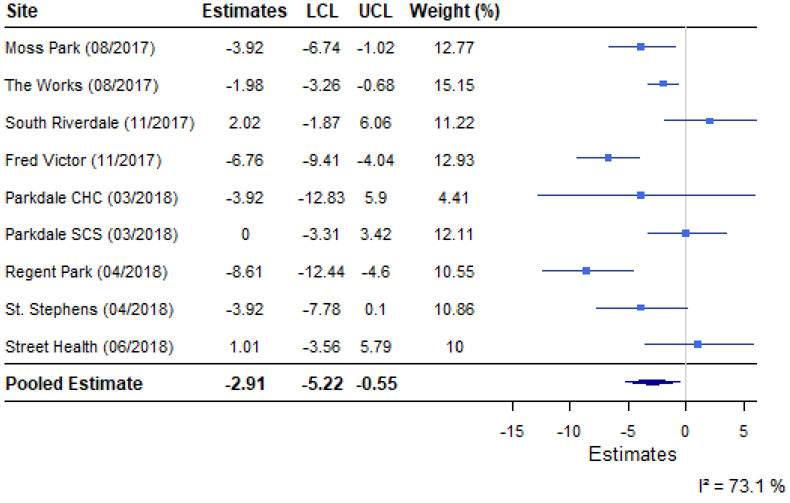

Pooled Level Estimate: Thefts from Vehicles - 400m

Site	Estimates	LCL	UCL	Weight (%)
Moss Park (08/2017)	13.88	-52.83	174.95	6.5
The Works (08/2017)	-19.75	-38.91	5.42	26.24
South Riverdale (11/2017)	-1	-68.82	214.39	4.07
Fred Victor (11/2017)	-38.74	-65.37	8.36	12.59
Parkdale CHC (03/2018)	-33.63	-72.78	61.79	6.38
Parkdale SCS (03/2018)	-66.71	-83.85	-31.39	8.93
Regent Park (04/2018)	27.12	-37.35	157.96	9.23
St. Stephens (04/2018)	12.75	-46.48	137.52	8.53
Street Health (06/2018)	9.42	-29.21	69.12	17.52
**Pooled Estimate**	**-17.11**	**-35.21**	**6.06**	

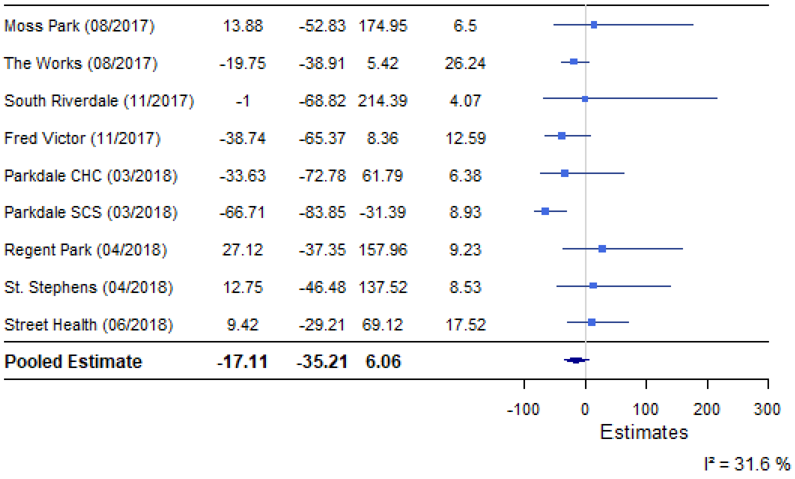

Pooled Trend Estimate: Thefts from Vehicles - 400m

Site	Estimates	LCL	UCL	Weight (%)
Moss Park (08/2017)	-2.96	-8.48	2.91	9.48
The Works (08/2017)	1.01	-1.09	3.15	12.47
South Riverdale (11/2017)	3.05	-1.27	7.55	10.89
Fred Victor (11/2017)	-3.92	-7.1	-0.63	11.63
Parkdale CHC (03/2018)	-12.19	-15.84	-8.38	10.91
Parkdale SCS (03/2018)	-1	-4.6	2.74	11.36
Regent Park (04/2018)	-1.98	-6.91	3.21	10.11
St. Stephens (04/2018)	4.08	0.08	8.24	11.18
Street Health (06/2018)	-6.76	-9.45	-4	11.96
**Pooled Estimate**	**-2.39**	**-5.7**	**1.03**	

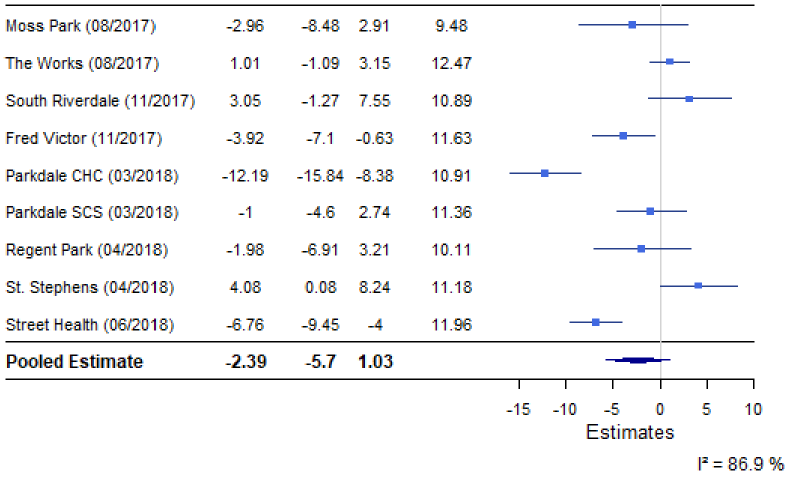

© 2025 Panagiotoglou D et al. *JAMA Network Open*.

eFigure 12. Percentage Changes in Level (Left) and Trend (Right) in Incidence of Assaults (Top) and Break and Enters (Bottom) Within 400m of OPS/SCS, Per Site and Pooled, Random Assignment

Pooled Level Estimate: Auto Theft - 400m

Site	Estimates	LCL	UCL	Weight (%)
Moss Park (08/2017)	29.69	-31.03	143.89	14.16
The Works (08/2017)	50.68	-31.83	233.05	12.08
South Riverdale (11/2017)	-38.12	-83.53	132.48	7.04
Fred Victor (11/2017)	375.88	-5.39	2293.64	5.34
Parkdale CHC (03/2018)	-63.94	-86.05	-6.76	10.29
Parkdale SCS (03/2018)	-1	-48.23	89.33	13.94
Regent Park (04/2018)	148.43	-0.8	522.18	10.63
St. Stephens (04/2018)	56.83	-20.8	210.56	13.47
Street Health (06/2018)	200.42	46.86	514.51	13.05
**Pooled Estimate**	**39.17**	**-9.95**	**115.06**	

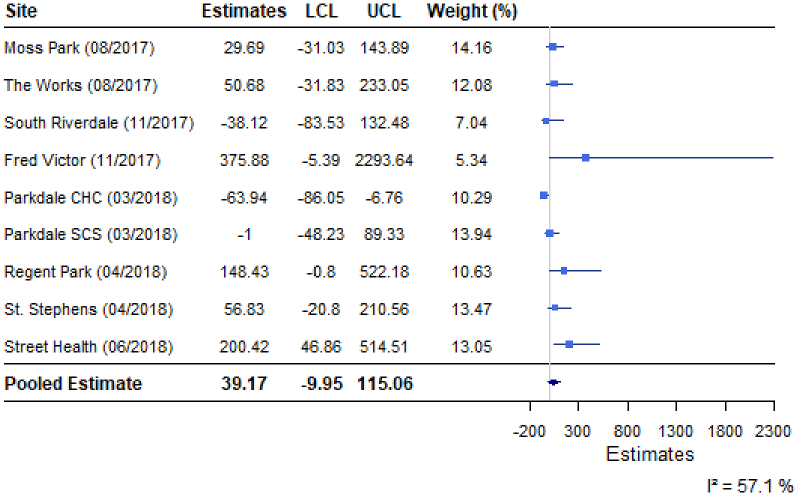

Pooled Trend Estimate: Auto Theft - 400m

Site	Estimates	LCL	UCL	Weight (%)
Moss Park (08/2017)	1.01	-0.83	2.87	16.2
The Works (08/2017)	-1	-3.4	1.47	9.05
South Riverdale (11/2017)	-4.88	-9.66	0.16	2.08
Fred Victor (11/2017)	2.02	-3.81	8.21	1.6
Parkdale CHC (03/2018)	-1	-2.82	0.86	15.68
Parkdale SCS (03/2018)	0	-1.68	1.71	18.75
Regent Park (04/2018)	1.01	-1.78	3.86	7.07
St. Stephens (04/2018)	0	-1.52	1.54	22.92
Street Health (06/2018)	-2.96	-5.71	-0.12	6.64
**Pooled Estimate**	**-0.29**	**-1.03**	**0.46**	

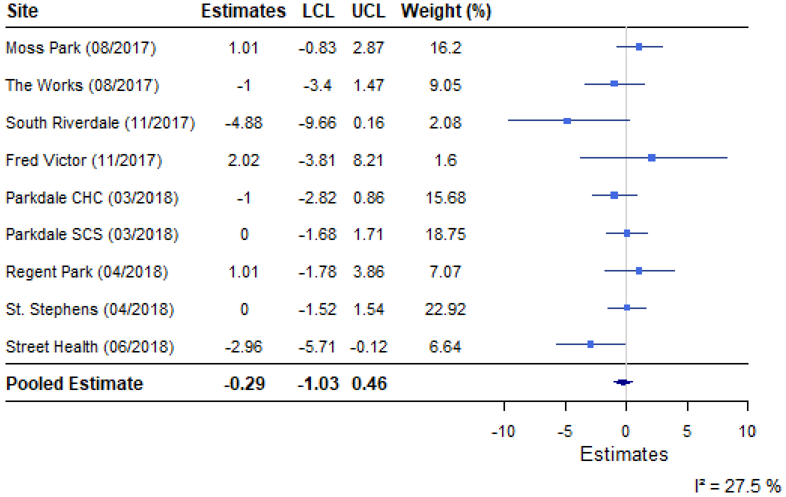

Pooled Level Estimate: B&E - 400m

Site	Estimates	LCL	UCL	Weight (%)
Moss Park (08/2017)	107.51	27.24	238.41	12.87
The Works (08/2017)	31	-13.15	97.59	16.71
South Riverdale (11/2017)	29.69	-29	136.89	9.16
Fred Victor (11/2017)	28.4	-36.69	160.43	6.94
Parkdale CHC (03/2018)	5.13	-39.83	83.66	10.41
Parkdale SCS (03/2018)	19.72	-20.93	81.27	16.49
Regent Park (04/2018)	93.48	-17.51	353.81	4.96
St. Stephens (04/2018)	177.32	53.07	402.43	9.37
Street Health (06/2018)	25.86	-22.38	104.09	13.1
**Pooled Estimate**	**45.28**	**19.15**	**77.13**	

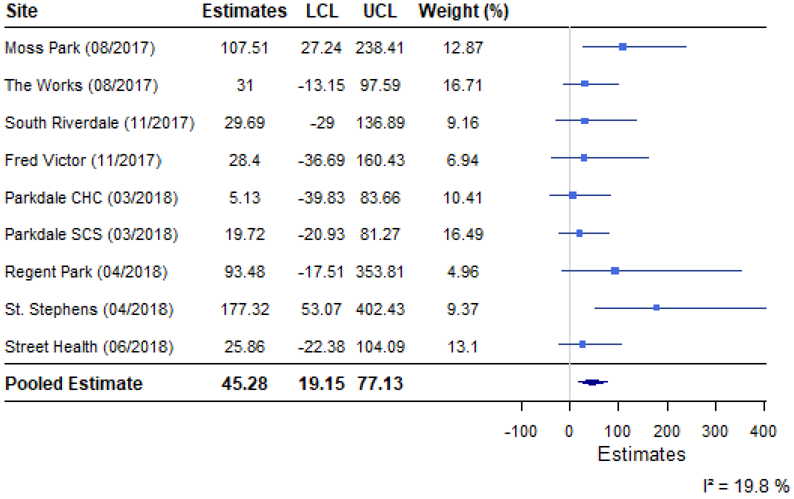

Pooled Trend Estimate: B&E - 400m

Site	Estimates	LCL	UCL	Weight (%)
Moss Park (08/2017)	0	-1.44	1.47	13.89
The Works (08/2017)	-1	-2.56	0.6	11.58
South Riverdale (11/2017)	-1	-2.68	0.72	10.04
Fred Victor (11/2017)	-1	-3.2	1.26	5.85
Parkdale CHC (03/2018)	-1	-2.35	0.38	15.52
Parkdale SCS (03/2018)	-1.98	-3.26	-0.68	16.84
Regent Park (04/2018)	0	-1.89	1.92	8.15
St. Stephens (04/2018)	-1.98	-3.62	-0.32	10.41
Street Health (06/2018)	-1.98	-3.88	-0.04	7.72
**Pooled Estimate**	**-1.12**	**-1.66**	**-0.58**	

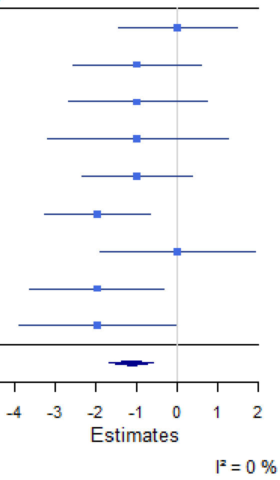

© 2025 Panagiotoglou D et al. *JAMA Network Open*.

eFigure 13. Percentage Changes in Level (Left) and Trend (Right) in Incidence of Robbery (Top) and Auto Theft (Bottom) Within 400m of OPS/SCS, Per Site and Pooled, Random Assignment

Pooled Level Estimate: Robbery - 400m

Site	Estimates	LCL	UCL	Weight (%)
Moss Park (08/2017)	52.2	6.53	117.44	15.65
The Works (08/2017)	6.18	-18.36	38.1	18.07
South Riverdale (11/2017)	-6.76	-52.98	84.87	8.77
Fred Victor (11/2017)	-43.45	-73.62	21.24	7.65
Parkdale CHC (03/2018)	61.61	-18.1	218.9	8.84
Parkdale SCS (03/2018)	-30.23	-64.34	36.51	8.98
Regent Park (04/2018)	-44.01	-72.76	15.06	8.23
St. Stephens (04/2018)	-45.12	-71	3.87	9.52
Street Health (06/2018)	10.52	-26.72	66.68	14.29
**Pooled Estimate**	**-5.22**	**-26.98**	**23.02**	

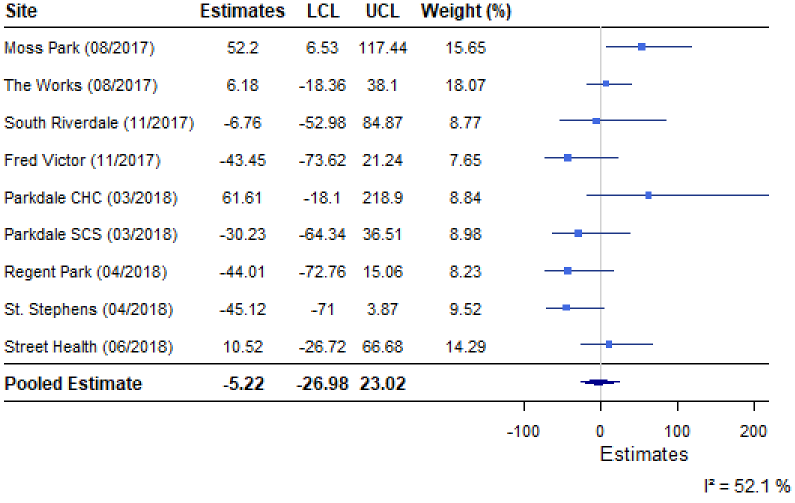

Pooled Trend Estimate: Robbery - 400m

Site	Estimates	LCL	UCL	Weight (%)
Moss Park (08/2017)	-1	-2.08	0.1	15.99
The Works (08/2017)	-1.98	-2.71	-1.24	21.59
South Riverdale (11/2017)	0	-1.93	1.97	7.82
Fred Victor (11/2017)	-1.98	-4.26	0.35	5.84
Parkdale CHC (03/2018)	-1	-3.07	1.12	6.91
Parkdale SCS (03/2018)	0	-1.54	1.56	10.82
Regent Park (04/2018)	-2.96	-4.87	-1	7.56
St. Stephens (04/2018)	0	-1.84	1.87	8.44
Street Health (06/2018)	-1	-2.15	0.17	15.03
**Pooled Estimate**	**-1.15**	**-1.77**	**-0.52**	

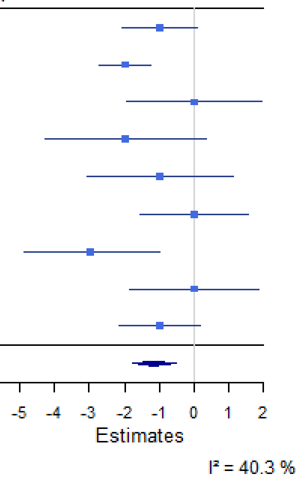

Pooled Level Estimate: Auto Theft - 400m

Site	Estimates	LCL	UCL	Weight (%)
Moss Park (08/2017)	29.69	-31.03	143.89	14.16
The Works (08/2017)	50.68	-31.83	233.05	12.08
South Riverdale (11/2017)	-38.12	-83.53	132.48	7.04
Fred Victor (11/2017)	375.88	-5.39	2293.64	5.34
Parkdale CHC (03/2018)	-63.94	-86.05	-6.76	10.29
Parkdale SCS (03/2018)	-1	-48.23	89.33	13.94
Regent Park (04/2018)	148.43	-0.8	522.18	10.63
St. Stephens (04/2018)	56.83	-20.8	210.56	13.47
Street Health (06/2018)	200.42	46.86	514.51	13.05
**Pooled Estimate**	**39.17**	**-9.95**	**115.06**	

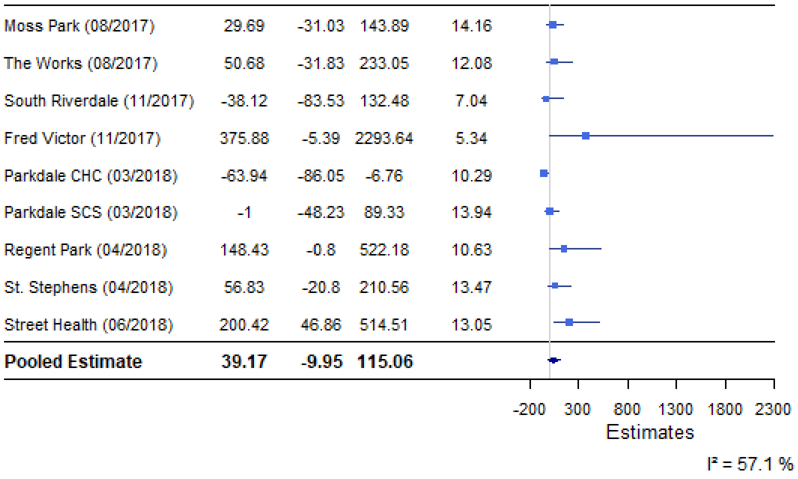

Pooled Trend Estimate: Auto Theft - 400m

Site	Estimates	LCL	UCL	Weight (%)
Moss Park (08/2017)	1.01	-0.83	2.87	16.2
The Works (08/2017)	-1	-3.4	1.47	9.05
South Riverdale (11/2017)	-4.88	-9.66	0.16	2.08
Fred Victor (11/2017)	2.02	-3.81	8.21	1.6
Parkdale CHC (03/2018)	-1	-2.82	0.86	15.68
Parkdale SCS (03/2018)	0	-1.68	1.71	18.75
Regent Park (04/2018)	1.01	-1.78	3.86	7.07
St. Stephens (04/2018)	0	-1.52	1.54	22.92
Street Health (06/2018)	-2.96	-5.71	-0.12	6.64
**Pooled Estimate**	**-0.29**	**-1.03**	**0.46**	

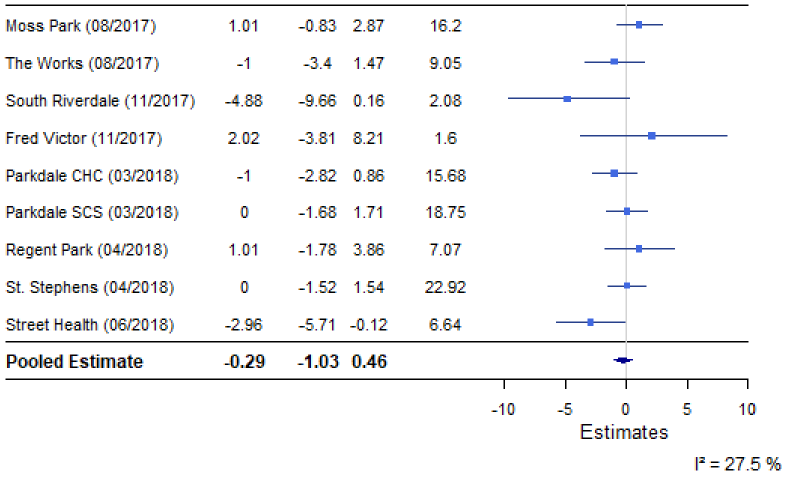

© 2025 Panagiotoglou D et al. *JAMA Network Open*.

eFigure 14. Percentage Changes in Level (Left) and Trend (Right) in Incidence of Thefts Over $5000 Within 400m of OPS/SCS, Per Site and Pooled, Random Assignment

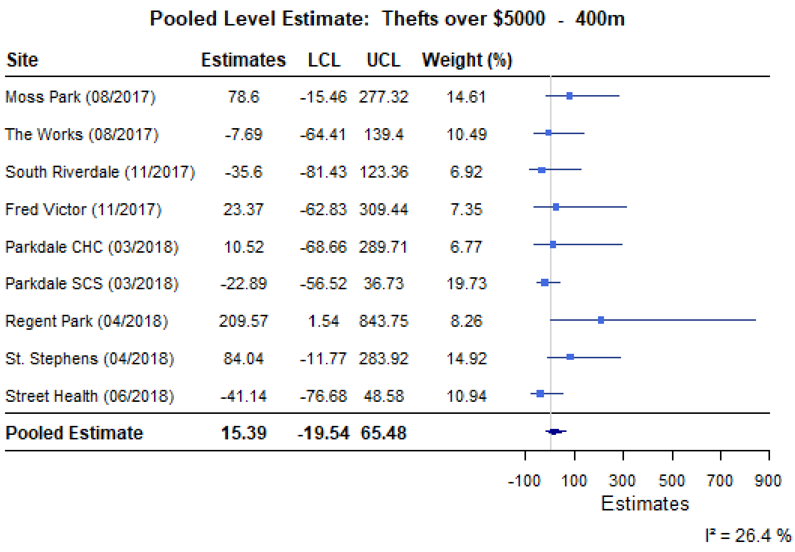

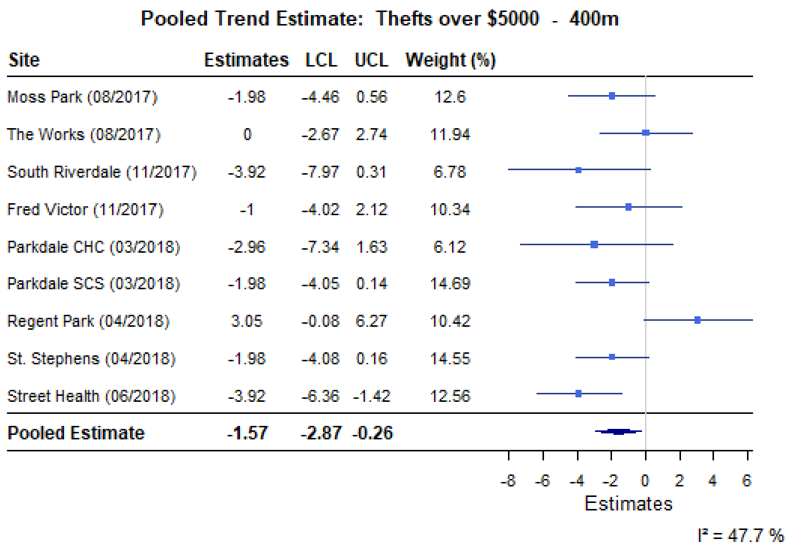

© 2025 Panagiotoglou D et al. *JAMA Network Open*.

eFigure 15. Percentage Changes in Level (Left) and Trend (Right) in Incidence of Bicycle Thefts (Top) and Thefts From Motor Vehicles (Bottom) Within 400m of OPS/SCS, Per Site and Pooled, Random Assignment

Pooled Level Estimate: Bicycle Thefts - 400m

Site	Estimates	LCL	UCL	Weight (%)
Moss Park (08/2017)	5.13	-18.73	36	13.99
The Works (08/2017)	37.71	-3.56	96.65	12.5
South Riverdale (11/2017)	-43.45	-64.56	-9.76	10.77
Fred Victor (11/2017)	31	-37.48	174.45	7.19
Parkdale CHC (03/2018)	-47.8	-70.13	-8.76	9.44
Parkdale SCS (03/2018)	-6.76	-35.01	33.76	12.43
Regent Park (04/2018)	-18.13	-46.61	25.54	11.38
St. Stephens (04/2018)	-31.61	-51.52	-3.54	12.69
Street Health (06/2018)	101.38	16.62	247.71	9.61
**Pooled Estimate**	**-6.65**	**-28.5**	**21.89**	

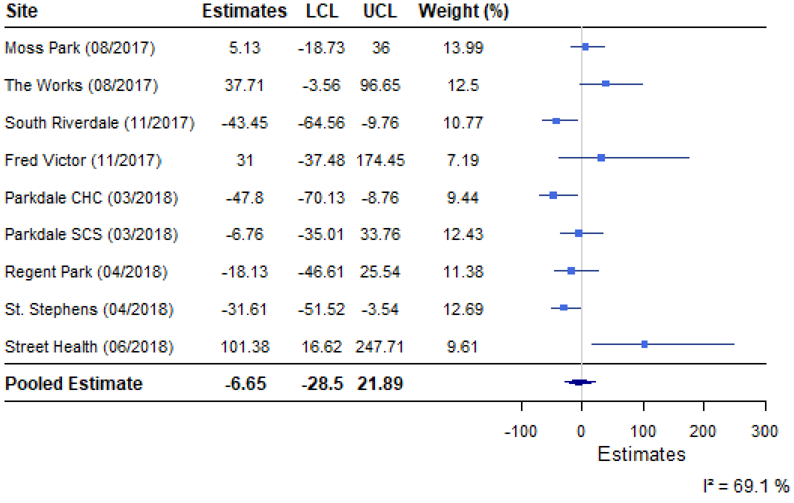

Pooled Trend Estimate: Bicycle Thefts - 400m

Site	Estimates	LCL	UCL	Weight (%)
Moss Park (08/2017)	-1.98	-2.95	-1	12.73
The Works (08/2017)	1.01	-0.38	2.41	11.45
South Riverdale (11/2017)	-1	-2.25	0.27	11.83
Fred Victor (11/2017)	-1.98	-3.98	0.07	9.09
Parkdale CHC (03/2018)	-3.92	-5.68	-2.13	9.84
Parkdale SCS (03/2018)	-1	-2.03	0.05	12.55
Regent Park (04/2018)	-3.92	-5.29	-2.53	11.25
St. Stephens (04/2018)	-1	-2.44	0.47	11.15
Street Health (06/2018)	-1	-2.72	0.77	10.12
**Pooled Estimate**	**-1.61**	**-2.61**	**-0.6**	

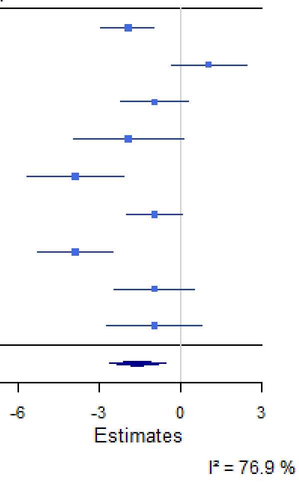

Pooled Level Estimate: Thefts over $5000 - 400m

Site	Estimates	LCL	UCL	Weight (%)
Moss Park (08/2017)	78.6	-15.46	277.32	14.61
The Works (08/2017)	-7.69	-64.41	139.4	10.49
South Riverdale (11/2017)	-35.6	-81.43	123.36	6.92
Fred Victor (11/2017)	23.37	-62.83	309.44	7.35
Parkdale CHC (03/2018)	10.52	-68.66	289.71	6.77
Parkdale SCS (03/2018)	-22.89	-56.52	36.73	19.73
Regent Park (04/2018)	209.57	1.54	843.75	8.26
St. Stephens (04/2018)	84.04	-11.77	283.92	14.92
Street Health (06/2018)	-41.14	-76.68	48.58	10.94
**Pooled Estimate**	**15.39**	**-19.54**	**65.48**	

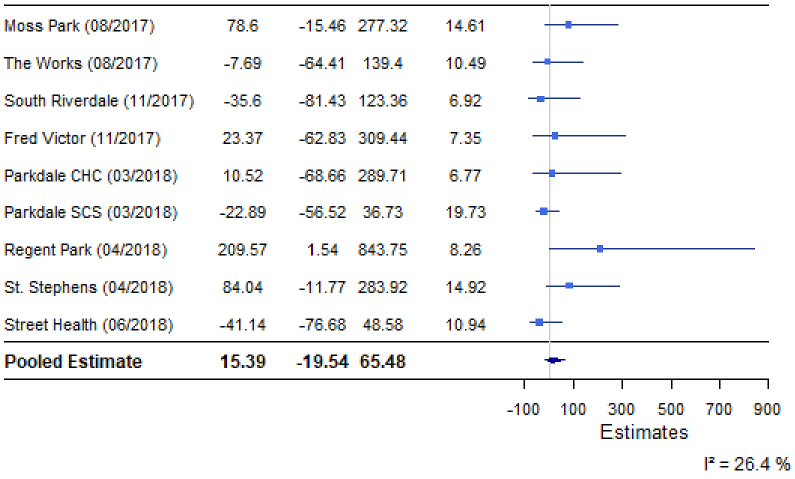

Pooled Trend Estimate: Thefts over $5000 - 400m

Site	Estimates	LCL	UCL	Weight (%)
Moss Park (08/2017)	-1.98	-4.46	0.56	12.6
The Works (08/2017)	0	-2.67	2.74	11.94
South Riverdale (11/2017)	-3.92	-7.97	0.31	6.78
Fred Victor (11/2017)	-1	-4.02	2.12	10.34
Parkdale CHC (03/2018)	-2.96	-7.34	1.63	6.12
Parkdale SCS (03/2018)	-1.98	-4.05	0.14	14.69
Regent Park (04/2018)	3.05	-0.08	6.27	10.42
St. Stephens (04/2018)	-1.98	-4.08	0.16	14.55
Street Health (06/2018)	-3.92	-6.36	-1.42	12.56
**Pooled Estimate**	**-1.57**	**-2.87**	**-0.26**	

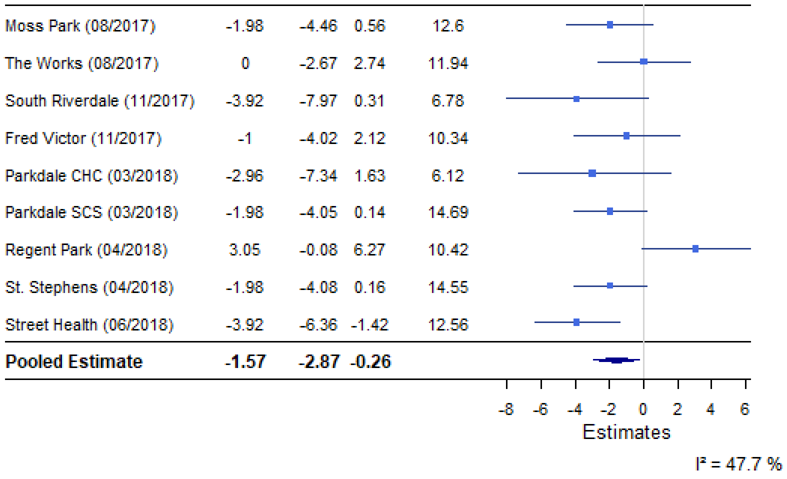

© 2025 Panagiotoglou D et al. *JAMA Network Open*.

eReferences
1. Statistics Canada. Classification of common offence. Accessed 26 September 2024, https://www23.statcan.gc.ca/imdb/p3VD.pl?Function=getVD&TVD=257740&CVD=257742&CPV=1.1&CST=01012015&CLV=1&MLV=3&D=1

© 2025 Panagiotoglou D et al. *JAMA Network Open*.
