# Supplementary material for: Toronto’s Supervised Consumption Sites and Local Crime
Source: JAMA Netw Open. 2025 Nov 25;8(11):e2545352. doi: 10.1001/jamanetworkopen.2025.45352 (PMC12648347; doi:10.1001/jamanetworkopen.2025.45352)
Supplement: Supplement 2. — Data Sharing Statement [file jamanetwopen-e2545352-s002.pdf]

Data Sharing Statement

Panagiotoglou. Toronto's Supervised Consumption Sites and Local Crime. *JAMA Netw Open*. Published November 25, 2025. doi:10.1001/jamanetworkopen.2025.45352

Data
Data available: Yes
Data types: Other (please specify)
Additional Information: This study uses publicly available data provided by Toronto Police Services and Environment Canada. We indicate in the manuscript where readers can access the data, and can provide the code for how we cleaned and analyzed the data upon request.
How to access data: This study used publicly available data provided by Toronto Police Services. For more information, please visit the Toronto Police Service Public Safety Data Portal at: https://data.torontopolice.on.ca/. For Environment Canada weather data please visit: https://climate.weather.gc.ca/historical_data/search_historic_data_e.html.
When available: With publication

Supporting Documents
Document types: Statistical/analytic code
How to access documents: For statistical code, please contact Jihoon Lim <jihoon.lim@rimuhc.ca>.
When available: With publication

Additional Information
Who can access the data: Anyone requesting the data.
Types of analyses: N/A
Mechanisms of data availability: Without investigator support
